# Primary intrathoracic liposarcomas: A clinicopathologic and molecular study of 43 cases in one of the largest medical centers of China

**DOI:** 10.3389/fonc.2022.949962

**Published:** 2022-08-17

**Authors:** You Xie, Wenyi Jing, Wei Zhao, Ran Peng, Min Chen, Ting Lan, Heng Peng, Xin He, Huijiao Chen, Zhang Zhang, Hongying Zhang

**Affiliations:** ^1^ Department of Pathology, West China Hospital, Sichuan University, Chengdu, China; ^2^ Department of Pathology, Sichuan Cancer Hospital and Institute, Sichuan Cancer Center, Cancer Hospital Affiliate to School of Medicine, University of Electronic Science and Technology of China, Chengdu, China

**Keywords:** liposarcoma, thorax, molecular analysis, well-differentiated liposarcoma, dedifferentiated liposarcoma, myxoid pleomorphic liposarcoma

## Abstract

**Introduction:**

Primary intrathoracic liposarcoma is extremely rare, and most published series lack genetic analyses. The aim of our study is to better understand the clinicopathologic and genetic features of these rare lesions.

**Materials and methods:**

Forty-three primary intrathoracic liposarcomas were identified and most cases were analyzed by systematic genetic studies, including fluorescence *in situ* hybridization (FISH), whole-exome sequencing (WES), and Sanger sequencing.

**Results:**

This series included 27 males and 16 females (ratios, 1.68:1) aged 24-73 years (median, 53 years). Tumors mainly occurred in the mediastinum (n=23, 53.5%), followed by pleural cavity (n=16, 37.2%) and lung (n=4, 9.3%). The study included 21 well-differentiated liposarcomas (WDLs), 19 dedifferentiated liposarcomas (DDLs), 2 myxoid pleomorphic liposarcomas (MPLs) and 1 pleomorphic liposarcoma (PL), without identification of myxoid liposarcoma. FISH analysis identified *MDM2* amplification in 17 of 18 WDLs (94.4%) and all DDLs (16/16, 100.0%). The *MDM2*-nonamplified WDL was *CDK4*-nonamplified but *FRS2*-amplified. WES and Sanger sequencing found somatic *TP53* mutation in the 2 MPLs. Follow-up information was available for 33 of 38 cases (86.8%). Thirteen patients (39.4%) showed no evidence of disease, 10 patients (30.3%) were alive with disease, and 8 patients (24.2%) died of disease. Fourteen cases developed recurrence and 1 with metastasis.

**Conclusions:**

WDL/DDL was the overwhelming subtype in this location, followed by MPL and PL. Analysis of the *FRS2* gene, in combination with *MDM2* and other genes of 12q13-15, may more precisely characterize WDL/DDLs. MPL is the most fatal subtype of this site. Further studies are needed to explore the role of *TP53* in the pathogenesis of MPL.

## Introduction

Liposarcoma is one of the most common soft tissue malignancies. The recent fifth World Health Organization (WHO) Classification of Tumors of Soft Tissue and Bone divided liposarcoma into 4 major clinicopathologic and genetic subtypes: atypical lipomatous tumor (ALT)/well-differentiated liposarcoma (WDL)/dedifferentiated liposarcoma (DDL), myxoid liposarcoma (ML), pleomorphic liposarcoma (PL), and myxoid pleomorphic liposarcoma (MPL) (1–5). ALT/WDL/DDL accounts for 50-60% of all liposarcomas and is characterized by the amplification of 12q13-15, including several oncogenes, such as *MDM2*, *CDK4*, *FRS2*, *HMGA2*, and *CPM*. The term “ALT” is used for tumors located in the site where surgical excision can be carried out and is curative, and the term “WDL” is used for lesions arising in sites such as the retroperitoneum, spermatic cord, and mediastinum, where tumors have a greater possibility for progression (1, 2). Nearly 20-30% of liposarcomas are MLs, most of which are characterized by the *FUS*-*DDIT3* fusion gene and a subset of tumors harboring the *EWSR1*-*DDIT3* fusion gene (3). PL represents less than 5% of liposarcoma with complex chromosomal aberrations (4). The newly proposed subtype, MPL, is an extremely rare and highly aggressive tumor (5).

Most liposarcomas occur in deep soft tissue of the extremities, followed by the retroperitoneum and trunk, although the location of liposarcomas depends on their subtype. Primary intrathoracic liposarcoma is very rare, accounting for only 1-2% of all liposarcomas (6). To the best of our knowledge, there were only 7 relatively large series of primary intrathoracic liposarcoma in the English literature, with clinical and pathologic information (7–13). However, the majority of previous series studies only reported mediastinal liposarcoma, without pleural and pulmonary tumors. Moreover, most series lacked systematic genetic studies, and only two previous large series explored genetic changes by fluorescence *in situ* hybridization (FISH) in 2/18 (11.1%) and 10/24 (41.7%) of their cases, respectively (10, 12).

Therefore, to better understand the clinicopathologic and genetic features of these rare lesions, we report a large series of 43 cases of primary intrathoracic liposarcomas from one of the largest medical centers in China. To the best of our knowledge, this is the largest population-based analysis with the highest rate for molecular testing in the English literature. Genetic analyses, including FISH, whole-exome sequencing (WES), and Sanger sequencing, were carried out in most cases (>90%).

## Materials and methods

### Case identification

This study was approved by the West China Hospital Institutional Review Board. A SNOMED search of hospital surgical pathology files from January 2007 to June 2021 identified 1386 liposarcoma cases. Seventy-eight liposarcomas were identified as intrathoracic liposarcomas, of which 35 tumors were metastatic or extended from other locations. All metastatic intrathoracic liposarcomas, primary cardiac liposarcomas, and intimal sarcomas arising in large blood vessels were excluded from the study. Finally, forty-three (43/1386, 3.1%) were included as primary intrathoracic liposarcomas in this study.

### Radiology methods

All available images of the cases were reviewed by one radiologist with thoracic tumor imaging expertise. Several parameters were evaluated, consisting of tumor margin and heterogeneity, pleural effusion, calcification, necrosis, cystic change and the involvement of other organs.

### Histologic evaluation

All cases were reviewed by 2 pathologists (H.Z., H.C.) with soft tissue tumor pathology expertise and 3 general surgical pathologists (Y.X., W.J., W.Z.) according to well-described criteria. Grading was evaluated following the ‘modified’ French Federation of Cancer Centers (FNCLCC) grading system (14).

### Immunohistochemistry (IHC)

Immunohistochemical analysis was performed on formalin-fixed, paraffin-embedded tissue using the Dako Envision Plus detection system (Dako, Carpinteria, CA, USA) with controls. The antibodies used included MDM2 (clone SMP14, ready-to-use; Abcam, Cambridge, UK), CDK4 (clone EP180, 1:100; Santa Cruz Biotechnology, Santa Cruz, CA, USA), S-100 protein (clone 4C49, 1:100; Abcam, Cambridge, UK), CD34 (clone QBEnd 10, 1:100; Abcam, Cambridge, UK), desmin (clone D33, 1:100; Dako, Carpinteria, CA, USA), smooth muscle actin (SMA) (clone 1A4, 1:100; Dako, Carpinteria, CA, USA), H-caldesmon (clone h-CD, 1:100; Dako, Carpinteria, CA, USA), and p53 (clone Do-7, ready-to-use; Dako, Carpinteria, CA, USA).

### Fluorescence *in situ* hybridization (FISH)

FISH analysis was conducted on formalin-fixed, paraffin-embedded 4-µm-thick tissue sections of 41 cases that had material available for further study. Additionally, FISH for the *CDK4* and *FRS2* was performed on the *MDM2* nonamplified case but with a potential diagnosis of WDL. *MDM2*, *CDK4*, *FRS2*, *DDIT3*, *HMGA2*, and *RB1* FISH analyses were performed using the commercially available Vysis *MDM2* Dual Color Probe (Abbott Molecular, Des Plaines, IL, USA), GSP *CDK4* (12q14) Gene Amplification Probe (Anbiping, Guangzhou, China), GSP *FRS2* (12q15) Gene Amplification Probe (Anbiping, Guangzhou, China), Vysis LSI *DDIT3* Dual Color Break Apart Probe (Abbott Molecular, Des Plaines, IL, USA) and Vysis LSI 13 (*RB1*) 13q14 Spectrum Orange Probe (Abbott Molecular, Des Plaines, IL, USA). All FISH analyses were performed according to a previously established laboratory protocol (15–17). Each case was examined and evaluated by counting a minimum of 100 nuclei by two independent investigators (H.Z. and M.C.). *MDM2*, *CDK4* or *FRS2* amplification was defined as an *MDM2/*CEP12, *CDK4/*CEP12 or *FRS2/*CEP12 ratio ≥2.0, and a ratio <2.0 was considered nonamplified. *DDIT3* gene rearrangement was defined as ≥ 10% cells exhibiting the split signal pattern, that is, the distance between the green and red signals was greater than the diameter of two signals. Cells containing *RB1* deletion displayed only one orange signal pattern. *RB1* deletion was defined as more than 25% of the cells exhibiting the deletion pattern.

### Whole exome sequencing (WES)

WES was performed on one MPL case (case 42) by Genomic OE Biotech Co., Ltd. (Shanghai, China). Genomic DNA was extracted using a QIAamp DNA Mini Kit (Qiagen, Valencia, CA, USA), and the quantification and integrity of DNA were identified by a Nanodrop spectrophotometer (Thermo Fisher Scientific, Inc., Wilmington, DE, USA). Genomic DNA samples were captured on an Agilent SureSelect whole exome library following the manufacturer’s protocol. In brief, genomic DNA was sheared, purified immediately and ligated with adapters. The amplification of the libraries was conducted by polymerase chain reaction (PCR) and then hybridized with custom probes. The bound DNA fragments were washed and eluted; then, these libraries were sequenced on the Illumina sequencing platform (HiSeq X-10, Illumina, Inc., San Diego, CA, USA), and 150 bp paired-end reads were generated.

### *TP53* mutation analysis

*TP53* mutation analysis was performed on the 2 MPL cases (including tumors and their adjacent normal tissues) by PCR and Sanger sequencing according to a previously reported method (15). Primers were used as follows: *TP53*-F: TCCCAAGCAATGGATGATTT, *TP53*-R: TTCTGGGAAGGGACAGAAGA. Sanger sequencing was performed by Tsingke Biological Technology Co., Ltd. (Chengdu, China).

### Statistical analysis

For survival analysis, overall survival (OS) was defined as time from disease diagnosis to death from tumor. DFS (disease-free survival) was defined as time from complete resection until local recurrence or metastasis. OS and DFS were analyzed using the Kaplan-Meier method followed with log- rank test. Data were analyzed using SPSS version 20.0 (IBM Corp, Armonk, NY, USA). *P* < 0.05 indicates the statistical significance between different groups.

## Results

### Clinical findings

The clinicopathologic findings of the 43 patients are summarized in **Table 1**. This study comprised 27 males and 16 females (ratios, 1.68:1) with a median age of 53 years (range, 24 to 73 years). The tumors involved the mediastinum (23/43, 53.5%), pleura space (16/43, 37.2%), and lung (4/43, 9.3%). Among the 23 mediastinal cases, 6 tumors involved anterior mediastinum (6/23, 26.1%), 4 in the posterior mediastinum (4/23, 17.3%), 3 in the superior mediastinum (3/23, 13.1%), and 3 tumors (3/23, 13.1%) extensively involved multiple mediastinal compartments. The remaining 7 (7/23, 30.4%) stated no definite mediastinal location.

**Table 1:** Clinicopathologic features of 43 primary intrathoracic liposarcomas.

Case No.	Age/sex	Symptoms	Size (cm)	Location	Histology	IHC results	Genetic results	Treatment	Outcome/Follow-up duration	
1	56/F	Cough, Short of breath	NA	Pleura space	WDL (lipoma-like)	ND	Failed	Marginal excision	AWD/113 mo	
2	43/M	Cough	NA	Mediastinum	WDL (lipoma-like)	ND	*MDM2*+(FISH)	Biopsy only	Lost	
3	65/M	Chest pain	6	Left pleura space	WDL (lipoma-like)	CDK4+	Failed	Complete excision	ANED/93 mo	
4	57/F	Cough	20	Right pleura space	WDL (lipoma-like)	ND	*MDM2*-, *CDK4*-, *FRS2* + (FISH)	Complete excision	ANED/16 mo	
5	70/F	Asymptomatic	26	Anterior mediastinum	WDL (lipoma-like) (original diagnosis: WDL)	MDM2+	*MDM2*+(FISH)	Marginal excision	Recurrence at 5 mo, 20 mo, and 53mo resected; DOD/70 mo	
6	43/F	Chest tightness,	12	Middle and posterior mediastinum	WDL (lipoma-like)	MDM2+, CDK4+	*MDM2*+(FISH)	Complete excision	ANED/48 mo	
7	64/M	Asymptomatic	22	Left pleura space	WDL (lipoma-like)	ND	*MDM2*+(FISH)	Complete excision	Recurrence at 31 mo, resected and RT; AWD/36 mo	
8	42/M	Asymptomatic	18	Anterior mediastinum	WDL (lipoma-like) with myxoid change	MDM2+, CDK4+	*MDM2*+(FISH)	Complete excision	Lost	
9	69/M	Asymptomatic	23	Posterior mediastinum	WDL (sclerosing and lipoma-like) with myxoid change	MDM2+, CDK4+	*MDM2*+(FISH)	Complete excision	ANED/35 mo	
10	73/F	Cough	20	Right pleura space	WDL (inflammatory and lipoma-like) (original diagnosis: WDL)	MDM2+, CDK4+	*MDM2*+(FISH)	Marginal excision	Recurrence at 37 mo, 61 mo and 72 mo, resected; DOD/72 mo	
11	50/M	Asymptomatic	12	Superior mediastinum (the right side)	WDL (lipoma-like) with myxoid change (original diagnosis: liposarcoma)	ND	*MDM2*+(FISH)	Marginal excision	Recurrence at 84 mo, resected and RT; AWD/161 mo	
12	64/F	Asymptomatic	6.4	Anterior mediastinum	WDL (lipoma-like) (original diagnosis: DDL with myxofibrosarcoma-like differentiation)	MDM2+, CDK4+	*MDM2*+, no *RB1* loss (FISH)	Marginal excision	Recurrence at 22 mo, resected; NED/42 mo	
13	46/F	Short of breath	NA	Pleura space (the whole)	WDL (lipoma-like) with myxoid change (original diagnosis spindle cell liposarcoma, spindle cell lipoma)	MDM2+, CDK4+	*MDM2*+, no *RB1* loss (FISH)	Marginal excision	Recurrence at 32 mo, resected; AWD/39 mo	
14	38/F	Asymptomatic	NA	Anterior mediastinum	WDL (lipoma-like)	ND	*MDM2*+(FISH)	Marginal excision; CT, RT	Recurrence at 33 and 66 mo, resected; AWD/120 mo	
15	65/M	Asymptomatic	11	Superior mediastinum (the right side)	WDL (inflammatory), with myxoid change	ND	ND	Complete excision	DFU/53 mo	
16	56/M	Asymptomatic	10.5	Left pleura space (involving to lung)	WDL (inflammatory) (original diagnosis inflammatory pseudotumor)	MDM2+, CDK4+	*MDM2*+(FISH)	Complete excision	ANED/37 mo	
17	53/M	Cough, Expectoration	17	Posterior mediastinum	WDL (sclerosing and lipoma-like)	MDM2+, CDK4+	*MDM2*+(FISH)	Marginal excision	AWD/17 mo	
18	37/F	Cough	12	Posterior mediastinum	WDL (sclerosing and lipoma-like)	MDM2+, CDK4+	*MDM2*+(FISH)	Marginal excision	Recurrence at 36 mo, RT+CT; AWD/43 mo	
19	57/M	Asymptomatic	19.2	Right and posterior mediastinum	WDL (sclerosing and lipoma-like) with myxoid change	MDM2+, CDK4+	*MDM2*+(FISH)	Complete excision	ANED/42 mo	
20	44/M	Short of breath	35	Posterior mediastinum	WDL (sclerosing and lipoma-like)	MDM2+, CDK4+	*MDM2*+(FISH)	Marginal excision; CT, RT	Recurrence at 16 and 28 mo, resected; DOD/36 mo	
21	38/M	Cough	10	Mediastinum	WDL (lipoma-like)	MDM2+, CDK4+	*MDM2*+, *CDK4*+, *FRS2* + (FISH)	Complete excision	ANED/7 mo	
22	62/M	Facial edema	16	Mediastinum	DDL, with undifferentiated pleomorphic sarcoma-like differentiation and myxoid change; well-differentiated liposarcoma area (lipoma-like) (original diagnosis: malignant tumor: 1.SFT 2. MFH 3. liposarcoma)	CDK4+	failed	Marginal excision	Lost	
23	48/M	Asymptomatic	NA	Mediastinum	DDL, with undifferentiated pleomorphic sarcoma-like differentiation and myxoid change; (original diagnosis: DDL)	MDM2+, CDK4+	*MDM2*+(FISH)	Marginal excision; CT, RT	DOD/3 mo	
24	56/M	Asymptomatic	18	Right pleura space	DDL, with high-grade myxofibrosarcoma- like differentiation; well-differentiated liposarcoma area (sclerotic); (original diagnosis: spindle cell tumor. 1. SFT with malignant transformation2. synovial sarcoma 3. thymoma (type A) 4. DDL need to be excluded)	MDM2+	*MDM2*+(FISH)	Complete excision	Lost	
25	58/M	Chest tightness, Short of breath	20	Anterior mediastinum	DDL, with undifferentiated pleomorphic sarcoma-like differentiation; well-differentiated liposarcoma area (sclerotic)	ND	ND	Marginal excision	Lost	
26	62/M	Cough, Expectoration, Chest pain	8	Inferior and anterior mediastinum	DDL, with undifferentiated pleomorphic sarcoma-like differentiation; well-differentiated liposarcoma area (sclerotic)	MDM2+, CDK4+	*MDM2*+(FISH)	Marginal excision; CT	Recurrence at 11 mo, resected; DOD/39 mo	
27	65/M	Short of breath	5	mediastinum	DDL, with undifferentiated pleomorphic sarcoma-like differentiation; well-differentiated liposarcoma area (sclerotic); (original diagnosis poorly differentiated sarcoma)	MDM2+, CDK4+	*MDM2*+(FISH)	Marginal excision	DOD/4 mo	
28	30/M	Chest pain	16	Left pleura space	DDL, with high-grade myxofibrosarcoma -like differentiation, well- differentiated liposarcoma area (lipoma-like and sclerotic)	MDM2+, CDK4+	*MDM2*+(FISH)	Complete excision, CT	ANED/24 mo	
29	53/M	Cough	24	mediastinum	DDL, with osteosarcoma/chondrosarcomatous-like differentiation and myxoid change, well- differentiated liposarcoma area (lipoma-like and sclerotic)	MDM2+, CDK4+	*MDM2*+, *DDIT3*- (FISH)	Complete excision	ANED/23 mo	
30	60/F	Asymptomatic	NA	Left lung tissue	DDL, with high-grade myxofibrosarcoma -like differentiation	MDM2+, CDK4+	*MDM2*+(FISH)	Biopsy; CT, RT	AWD/12 mo	
31	53/F	Cough, Expectoration, Dorsagia	12	Left pleura space	DDL, with myxofibrosarcoma-like differentiation and myxoid change, well-differentiated liposarcoma area (lipoma-like)	ND	*MDM2*+(FISH)	Marginal excision; CT	Recurrence at 25 and 37 mo, resected; AWD/55 mo	
32	43/M	Cough, Expectoration, Chest pain	20	Right pleura space	DDL, with myxofibrosarcoma-like differentiation and myxoid change, well- differentiated liposarcoma area (lipoma-like and sclerotic)	MDM2+,	*MDM2*+, *DDIT3*- (FISH)	Marginal excision, CT	Recurrence at 58 and 74 mo, Scalp metastasis at 70 mo, resected; AWD/91 mo	
33	44/M	Short of breath	7	Right pleura space (involving to lung tissue)	DDL with leiomyosarcomatous differentiation, well- differentiated liposarcoma area (sclerotic)	MDM2+, CDK4+, SMA+, Desmin+	*MDM2*+(FISH)	Complete excision	Lost	
34	62/M	Cough, Expectoration	1.2	Left lung tissue	DDL, with myxofibrosarcoma-like differentiation	CDK4+	*MDM2*+(FISH)	Complete excision	ANED/28 mo	
35	31/F	Cough, Expectoration	26.4	Right pleura space	DDL, with IMT-like differentiation, and myxoid change, well-differentiated liposarcoma area (lipoma-like)	MDM2+, CDK4+	*MDM2*+(FISH)	Complete excision	ANED/30 mo	
36	40/F	Cough, Chest pain	13	Left pleura space	DDL, with IMT-like differentiation, well-differentiated liposarcoma area (sclerotic)	ND	*MDM2*+(FISH)	Marginal excision; CT	DFU/17 mo	
37	40/F	Cough	10.2	Right pleura space	DDL, with low-grade fibrosarcoma-like differentiation (desmoid-type fibromatosis-like); well-differentiated liposarcoma area (sclerotic)	ND	ND	Marginal excision	AWD/13 mo	
38	72/F	Short of breath	NA	Right lung tissue	DDL, with IMT-like differentiation well-differentiated liposarcoma area (sclerotic); (original diagnosis: low-grade soft tissue tumor, with a tendency to histocytic tumor or hemanyiopericytoma)	ND	*MDM2*+(FISH)	Biopsy, CT	Lost	
39	46/M	Cough, Short of breath	35	Right pleura space	DDL, with low-grade fibrosarcoma-like differentiation, well- differentiated liposarcoma area (inflammatory and sclerotic)	MDM2+, CDK4+	*MDM2*+(FISH)	Marginal excision	Recurrence at 65 mo, resected; AWD/105 mo	
40	51/M	NA	NA	Lung	DDL with osteosarcomatous differentiation	ND	*MDM2*+(FISH)	Biopsy only	DOD/6 mo	
41	24/M	Asymptomatic	15	Superior mediastinum	MPL, displaying myxoid stroma, and pleomorphic lipoblasts (original diagnosis: desmoid-type fibromatosis)	P53+, MDM2-, CDK4-, CD34-, S100-	*MDM2*-, *DDIT3*-, no *RB1* loss (FISH); *TP53* somatic mutation (PCR)	Marginal excision, CT, RT	Recurrence at 3 mo, unresected; DOD/9 mo	
42	49/F	Dizziness, Hemoptysis, Chest tightness	7.8	Anterior mediastinum	MPL, displaying myxoid stroma, and pleomorphic lipoblasts; (original diagnosis: malignant tumor with the tendency to soft tissue sarcoma)	P53+, MDM2-, CDK4-, CD34-, S100-	*MDM2*-, *DDIT3*-, no *RB1* loss (FISH); *TP53* somatic mutation (WES&PCR)	Marginal excision	DOD/7 mo	
43	59/M	Short of breath	NA	mediastinum	PL, displaying spindle, pleomorphic tumors cells with pleomorphic lipoblasts	P53+, MDM2-	*MDM2*-, *CDK4*-, *FRS2*-(FISH)	Biopsy only	AWD/7 mo	

M, male; F, female; NA, not available; WDL, well-differentiated liposarcoma; DDL, de-differentiated liposarcoma; M-PL, myxoid pleomorphic liposarcoma; SFT, solitary fibrous tumor; MFH, malignant fibrous histiocytoma; IMT, inflammatory myofibroblastic tumor; IHC, immunohistochemistry; “+” positive, “-” negative; FISH, fluorescence *in situ* hybridization; PCR, polymerase chain reaction; WES, whole exome sequencing; RT, radiotherapy; CT, chemotherapy; ANED, alive with no evidence of disease; AWD, alive with disease; DFU, died from unrelated reasons; DOD, died of disease; ND, not done; mo, month.

### Radiologic findings

Computed tomography (CT) images were available in thirty-two patients (32/43, 74.4%). The tumor margins were well defined in 15 cases (15/32, 46.9%), ill-defined in 15 cases (15/32, 46.9%) and infiltrative in 2 cases (2/32,6.2%). Twenty-two cases had contrast-enhanced CT data, 16 of 22 (72.7%) cases displayed heterologous enhancement, and 6 cases (27.3%) showed homologous enhancement. Necrosis or cystic change was identified in 5 of 32 (15.6%) cases, and 1 of 32 cases (3.1%) had calcification. Seventeen cases (17/32, 53.1%) extensively involved or compressed the adjacent tissues and vital vessels, and pleural effusion was found in five cases (5/32, 15.6%). None of the 32 cases showed lymphadenopathy.

### Clinical treatments

Surgical excisions were performed on 38 patients (38/43, 88.4%), including marginal excision (n=22) and complete excision (n=16), and 9 patients (23.7%) received chemotherapy and/or radiotherapy. Five patients (5/43, 11.6%) received biopsy only, and 2 were treated with chemotherapy and/or radiotherapy.

### Pathologic and molecular findings

#### Gross findings

Macroscopic descriptions were available in 26 of the 38 (68.4%) resected specimens. Macroscopically, eighteen cases (18/26, 69.2%) were well-circumscribed masses, and the remaining 8 lesions (8/26, 30.8%) were poorly circumscribed with infiltration of adjacent organs or tissues. The cut surface of tumors showed a solid appearance from yellow to white.

### Microscopic and molecular findings

#### WDL (N=21)

**Figure 1:**
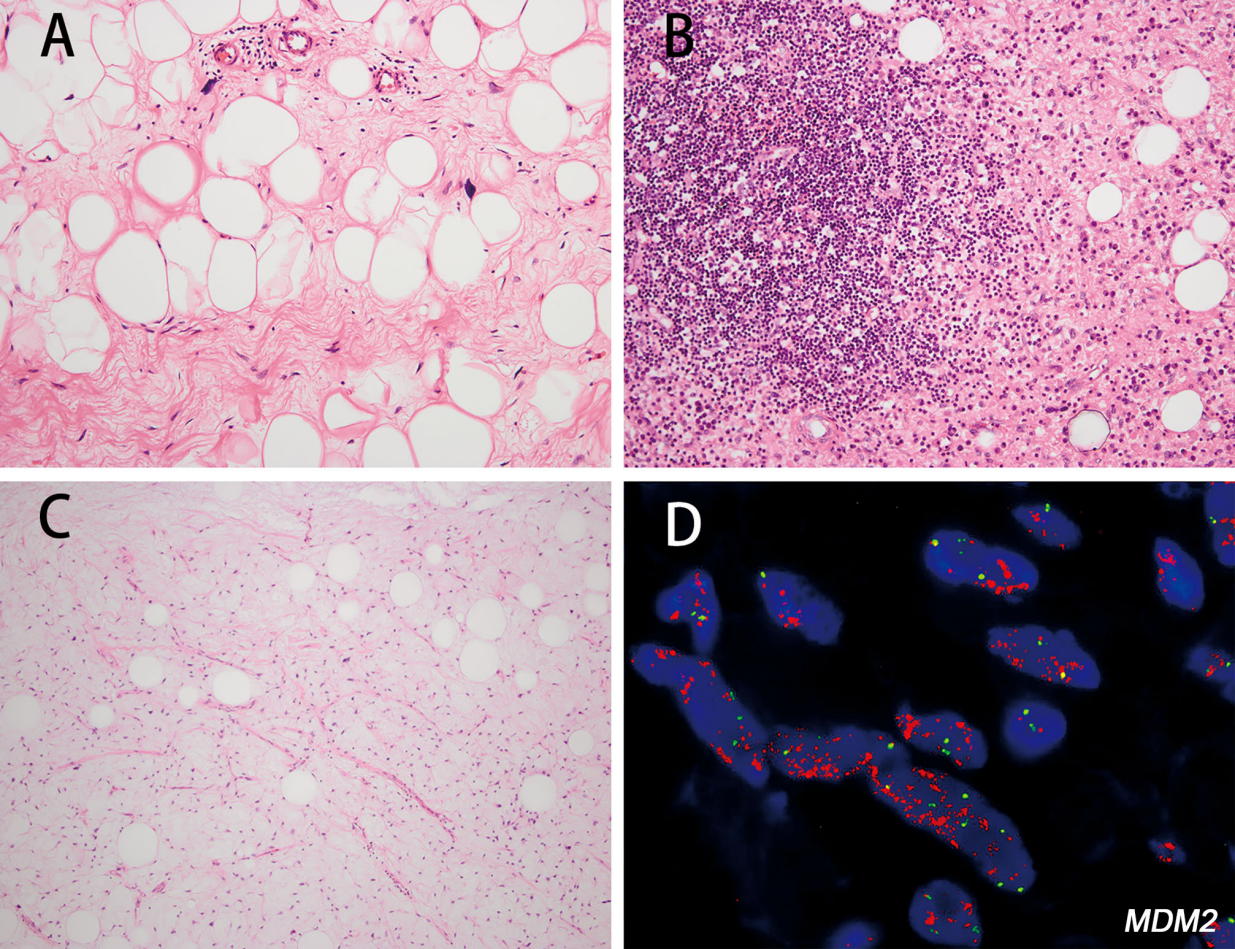
The histologic features of well-differentiated liposarcoma (WDL) and corresponding fluorescence *in situ* hybridization (FISH) images. Lipoma-like WDL showing variation in adipocyte size, with the presence of bizarre, hyperchromatic stromal cells (**A** hematoxylin and eosin staining [H&E]; magnification: 200×). Inflammatory WDL with predominant inflammatory cell arrogation and atypical, hyperchromatic cells can be identified in the stroma (**B** H&E; magnification: 200×). WDL with extensive myxoid change showing abundant myxoid stroma and containing small branching vessels (**C** H&E; magnification: 200×). FISH analysis identified *MDM2* amplification in the WDL (case 17) **(D)**.

The 21 conventional WDLs comprised 13 lipoma-like (61.9%) (**Figure 1A**), 2 inflammatory (9.5%) (**Figure 1B**), and 6 mixed-subtype tumors (28.6%). Lipoma-like WDL tumors were mainly composed of atypical adipocytes of varying sizes, and inflammatory WDL was characterized by extensive chronic inflammatory infiltrate. The 6 mixed-type tumors included 5 cases with mixed lipoma-like and sclerosing subtypes, and 1 case was a mixture of lipoma-like and inflammatory variants. Atypical, hyperchromatic stromal cells were identified in all of the tumors. A small focal area with increased cellularity was found in 10 cases, consistent with the morphology of cellular WDL. Myxoid change (**Figure 1C**) was found in 6 of 21 (28.6%) cases.h

**Figure 2:**
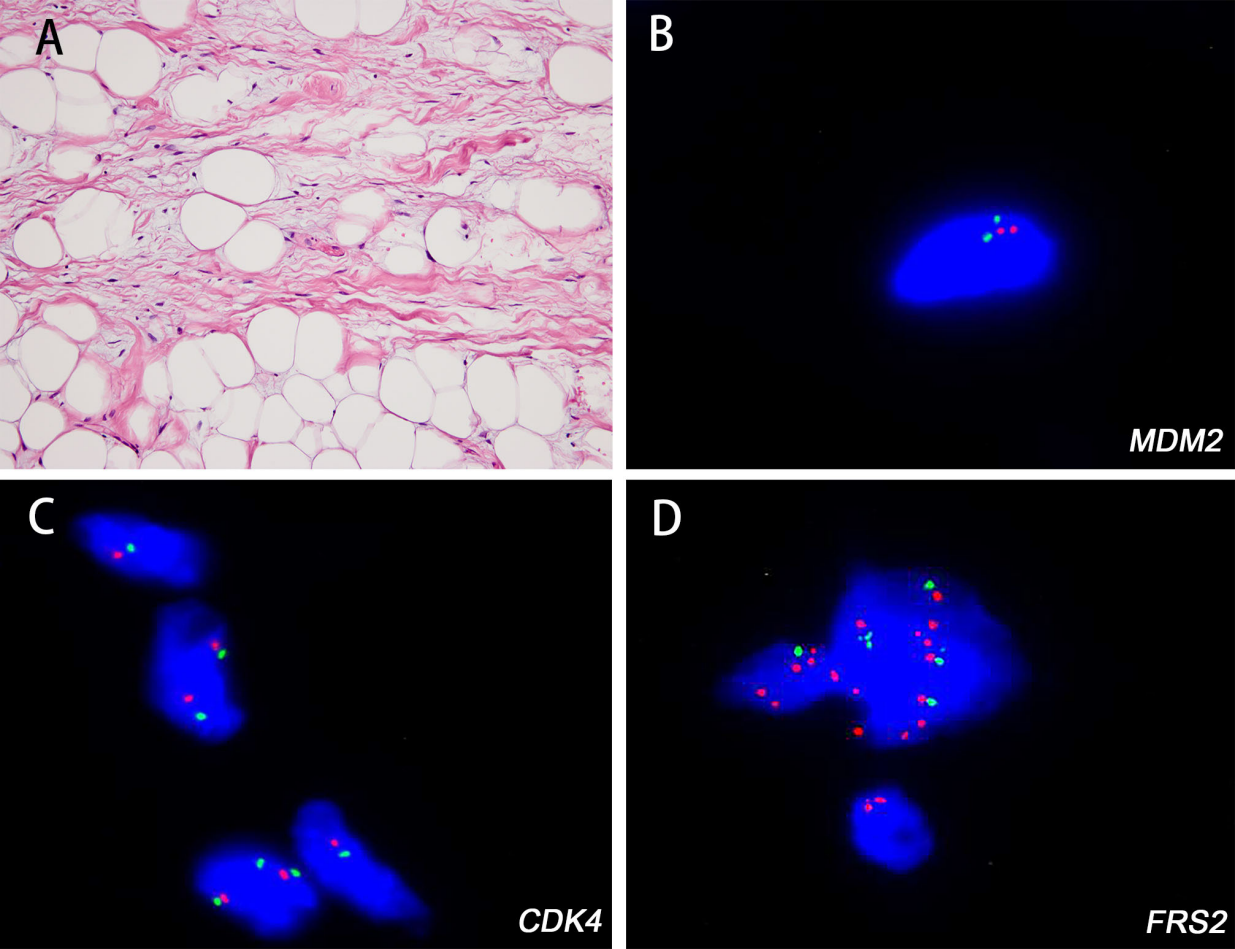
The histologic features of well-differentiated liposarcoma (WDL) with unusual genetic results and corresponding fluorescence *in situ* hybridization (FISH) images. The WDL (case 4) showing hyperchromatic bizarre stromal cells (**A** H&E; magnification: 200×). FISH analysis revealed that the tumor was negative for *MDM2* **(B)** and *CDK4* **(C)** gene amplification but with *FRS2* gene amplification **(D)**.

Immunohistochemically, MDM2 and CDK4 positivity was observed in 13/13 (100.0%) and 13/13 (100.0%) WDL cases, respectively. *MDM2* amplification was observed in 17/18 (94.5%) cases (**Figure 1D**). The *MDM2* FISH-negative case was *CDK4*-nonamplified but *FRS2*-amplified (case 4) (**Figures 2A–D**).

#### DDL (N=19)

Among the 19 DDLs, 15 cases (15/19, 79.0%) comprised WDL and DDL components simultaneously, and 4 cases only had DDL components. In the 15 cases with both components, 11 cases (11/15, 73.3%) showed an abrupt transition from WDL to DDL components, 3 cases (3/15, 20.0%) with gradual transition, and one case (1/15, 6.7%) with a mosaic transition pattern.

**Figure 3:**
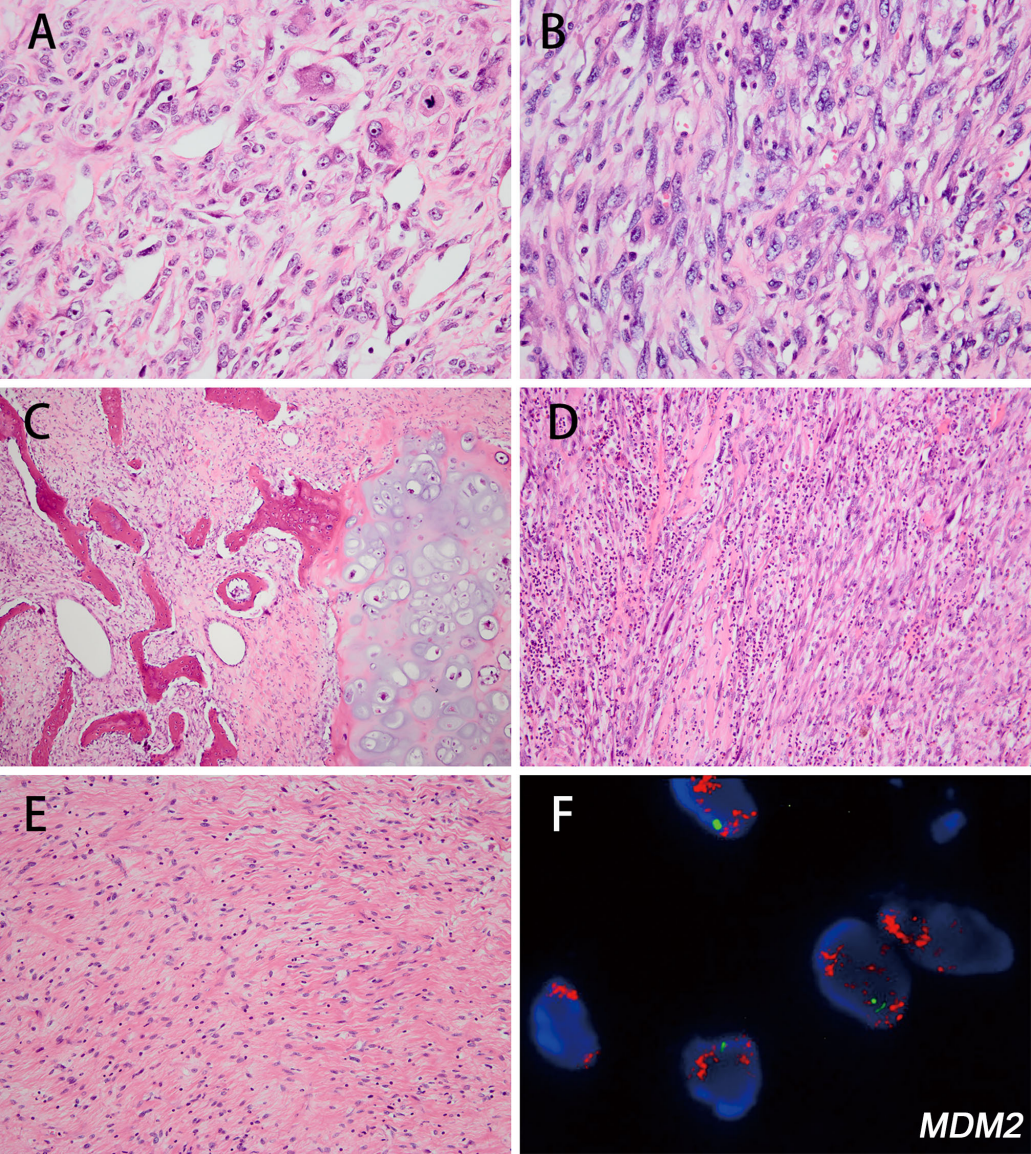
The histologic features of dedifferentiated liposarcoma (DDL) and corresponding fluorescence *in situ* hybridization (FISH) images. DDL with undifferentiated pleomorphic sarcoma-like differentiation; tumor cells exhibited moderate cytologic atypia with obvious nuclear pleomorphism (**A** H&E; magnification: 400×). DDL showed a fibrosarcoma-like pattern, exhibiting marked hypercellularity and cytologic atypia. (**B** H&E; magnification: 400×). DDL showing areas of osteosarcoma and chondrosarcoma-like differentiation (**C** H&E; magnification: 100×). DDL with IMT-like features with varying degrees of chronic inflammatory cell infiltration (**D** H&E; magnification: 200×). DDL with low-grade fibrosarcoma-like differentiation, exhibiting mild cytologic atypia (**E** H&E; magnification: 200×). FISH analysis identified *MDM2* amplification in the DDL (case 32) **(F)**.

In 19 DDL cases, 13 (68.4%) tumors exhibited classic histologic patterns, including undifferentiated pleomorphic sarcoma-like (n=5, 26.4%) (**Figure 3A**) and intermediate- to high-grade myxofibrosarcoma/fibrosarcoma-like patterns (n=6, 31.6%) (**Figure 3B**), and 2 cases exhibited osteosarcomatous/chondrosarcomatous differentiation (**Figure 3C**). The other 6 cases (31.6%) manifested uncommon dedifferentiated components, including 3 with inflammatory myofibroblastic tumor (IMT)-like morphology (15.8%), 2 cases with low-grade dedifferentiation (10.5%), and one DDL with leiomyosarcomatous differentiation. The mitotic rate ranged from 1-30 per 10 high-power fields (HPFs). Necrosis was found in 6 of 19 cases (31.6%). Myxoid change was identified in 6 cases (31.6%). Thirteen DDLs were classified as FNCLCC 2, and 6 DDLs were graded as FNCLCC 3.

In IMT-like DDLs (cases 35, 36, 38), the spindled tumor cells ranged in a fascicular cluster set in a slight myxoid matrix with varying degrees of chronic inflammatory cell infiltration. The spindle cells had abundant, eosinophilic cytoplasm and vesicular nuclei with small nucleoli (**Figure 3D**). Two DDL cases showed low-grade dedifferentiation resembling low-grade fibrosarcoma or desmoid fibromatosis (cases 37, 39). The tumor cells organized in a fascicular pattern, exhibiting moderate cellularity. The spindle cells had abundant, eosinophilic cytoplasm with mild nuclear atypia (**Figures 3E, F**).

**Figure 4:**
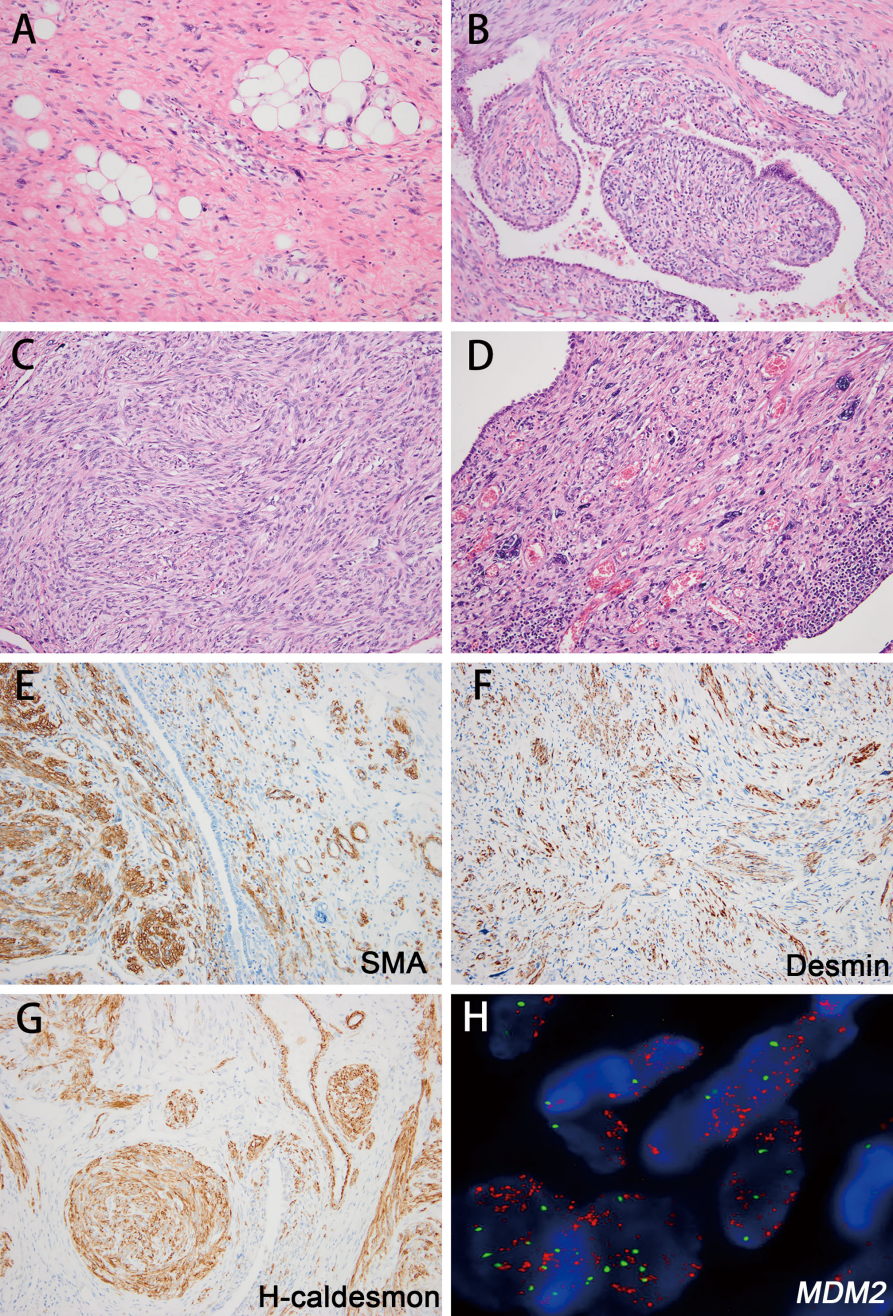
The histologic features of DDL with leiomyosarcomatous differentiation (case 33) and its corresponding immunohistochemical and fluorescence *in situ* hybridization (FISH) image. Sclerotic well-differentiated area outside the lung (**A** H&E; magnification: 200×). At low magnification, the growth of the spindle tumor cells showed cleft-like architecture, mimicking the pattern of pulmonary adenofibroma (**B** H&E; magnification: 200×). Spindle tumor cells display a fascicular arrangement, with hyperchromatic, cigar-shaped nuclei and mild to moderate atypia (**C** H&E; magnification: 200×). The tumor cells exhibited increased nuclear atypia with obvious pleomorphism (**D** H&E; magnification: 200×). The tumor cells showed SMA (**E** magnification: 200×), desmin (**F** magnification: 200×) and h-caldesmon (**G** magnification: 200×) positivity in well-differentiated areas and negativity in focal sarcoma-like areas. *MDM2* amplification was identified in this case **(H)**.

In one case (case 33), the tumor developed based on the pleura, involving the pleural cavity and lung parenchyma simultaneously. The pleural part was composed of a classic WDL component (**Figure 4A**). The lesion within the lung parenchyma exhibited more complexity and diversity. At low magnification, the growth of the spindle tumor cells under the bronchiolar epithelium caused cleft-like architecture, mimicking the pattern of pulmonary adenofibroma (**Figure 4B**). At high magnification, most areas displayed fascicular arrangement of spindle tumor cells, with hyperchromatic, cigar-shaped nuclei and mild to moderate atypia, mimicking low-grade smooth muscle tumors (**Figure 4C**). In the focal area of the lesion, the tumor cells exhibited increased nuclear atypia with obvious pleomorphism (**Figure 4D**). The mitotic activity increased to 12 per 10 HPF in this area, and atypical mitosis and necrosis could be identified.

Immunoreactivity for MDM2 and CDK4 was present in 11/11 (100%) and 11/11 (100%) DDL cases, respectively. The IHC results of DDL with well-differentiated leiomyosarcoma-like area (case 33) exhibited positivity for smooth muscle actin, desmin and h-caldesmon. In the dedifferentiated area, the tumor cells were negative for those myogenic markers (case 33; **Figures 4E–G**). High level *MDM2* amplification was identified in 16/16 cases (100%) (**Figure 4H**), including 2 cases with myxoid change, which were negative for *DDIT3* rearrangement (cases 29, 32).

#### MPL (N=2)

**Figure 5:**
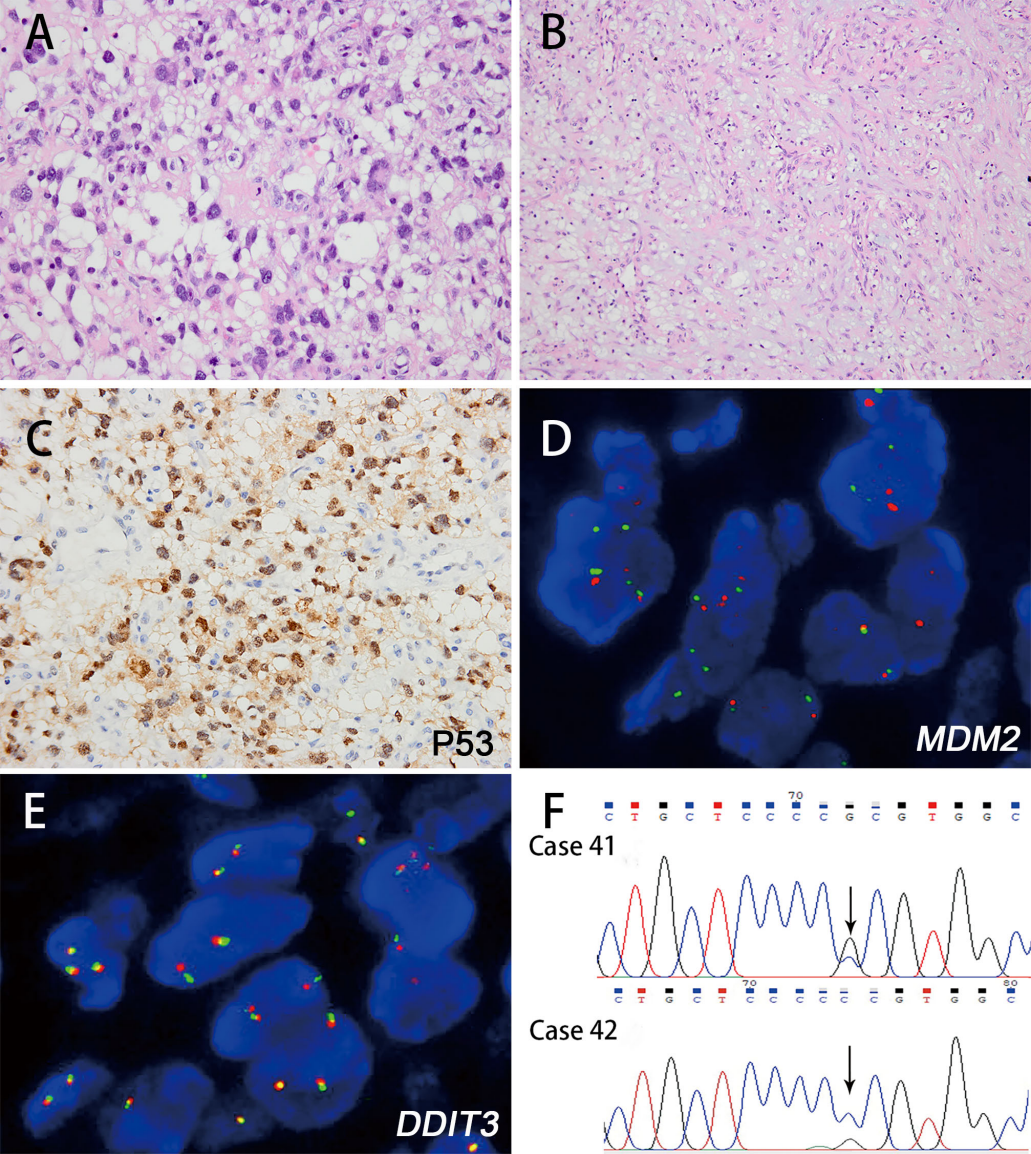
The histologic features of myxoid pleomorphic liposarcoma and corresponding immunohistochemical and genetic results. The tumor exhibited features of pleomorphic liposarcoma with myxoid matrix, and multivacuolated lipoblasts were found (**A** H&E; magnification: 400×) (case 42). Myxoid liposarcoma-like areas were also identified within the tumor, displaying a well-developed plexiform vasculature pattern (**B** H&E; magnification: 400×). The tumor cells (case 42) showed positivity for p53 immunostaining (**C** magnification: 400×). FISH analysis revealed negativity for *MDM2* amplification **(D)** or *DDIT3* rearrangement **(E)**. Sanger sequencing results showed *TP53* mutations in both tumors **(F)**.

Two cases (cases 41, 42) showed mixed features of conventional PL and variable portions of myxoid background (**Figure 5A**). The tumor cells in the PL-like area exhibited marked atypia, and pleomorphic lipoblasts were also identified. The conspicuous ML-like areas ranged from 40-60% in each case, displaying a well-developed plexiform vasculature pattern, pulmonary edema-like mucous pool, and scattered small lipoblasts and bland round cells (**Figure 5B**). The mitotic rates varied from 22 to 27 mitoses per 10 HPF. Necrosis could be observed in both cases. The 2 MPLs were graded as FNCLCC 3.

Both of the cases were strongly positive for p53(**Figure 5C**) and negative for CD34, MDM2, CDK4, and S-100 protein. FISH analysis revealed that both MPLs were negative for *MDM2* amplification (**Figure 5D**), *DDIT3* rearrangement (**Figure 5E**), and *RB1* deletion.

WES was performed on 1 MPL (case 42) to find more detailed genetic change of this subtype. The structural variants (SV) analysis showed that the number of interchromosomal translocation was the most frequent variant, followed by intrachromosomal translocation, deletion, duplication and inversion. Copy number variation (CNV) result showed the loss locus of chromosomes were more than the gains. The most frequent loss loci were 8p23.1, 16p13.11 and 1q21.3, and gain loci of 14q11.2, 7q22.1 and 7q11.23 were found. Somatic *TP53* mutation in exon 4: c.215G>C, p.P72R. was detected in this MPL, which was verified by Sanger sequencing analysis. The same result was observed in another MPL (case 41), while the *TP53* mutation was not detected in the nontumorous tissues of either case (**Figure 5F**).

#### PL (N=1)

**Figure 6:**
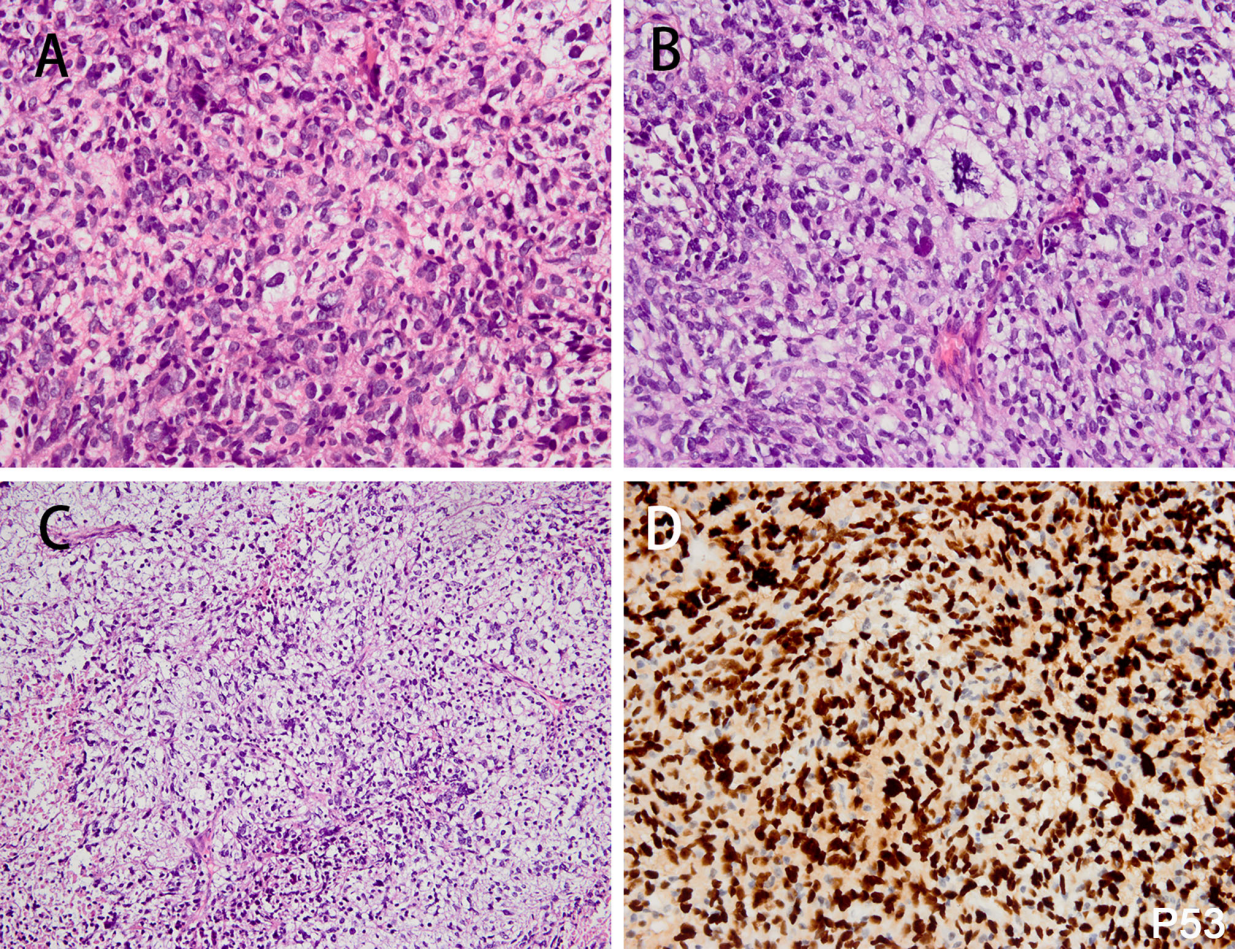
The histologic features of pleomorphic liposarcoma and corresponding immunohistochemical results. The spindled, epithelial tumor cells exhibited marked nuclear atypia (**A** H&E; magnification: 400×) (case 43). The bizarre lipoblasts can be identified within the lesion (**B** H&E; magnification: 400×). Myxoid change in focal area, resembling the morphology of myxofibrosarcoma (**C** H&E; magnification: 200×). The tumor cells were diffusely positive for P53 (**D** magnification: 400×).

The lesion comprised spindle, epithelioid tumor cells with severe atypia. The bizarre lipoblasts can be identified in some areas within the tumor (**Figures 6A, B**). Myxoid change was identified in focal area, resembling the morphology of myxofibrosarcoma (**Figure 6C**). Tumor necrosis and atypical mitotic figure were seen in the tumor. Immunohistochemical analysis revealed that the tumor cells were diffusely positive for P53 (**Figure 6D**) and focally positive for S-100 but negative for MDM2 protein. FISH analysis revealed that the tumor was negative for *MDM2*/*CDK4*/*FRS2* amplification.

### Clinical follow-up

Clinical follow-up data were available for 33 of 38 (86.8%) patients who received surgical intervention, with a median follow-up duration of 37 months (mean, 44.8 months; range 3 - 161 months). Local recurrence occurred in 14 cases (14/33, 42.4%) (median, 25 months; range 3- 84 months), including 9 WDLs, 4 DDLs and 1 MPL. Metastasis occurred in 1 patient with DDL. Thirteen patients (13/33, 39.4%) were alive with no evidence of disease (median, 35 months; range 7-105 months), including 8 WDLs and 5 DDLs. Ten patients (10/33, 30.3%) were alive with disease from 13 to 161 months after diagnosis (median, 49 months), including 7 WDL cases and 3 DDLs. Eight patients (8/33, 24.2%) died of disease, including 3 WDLs, 3 DDLs and 2 MPLs. Additionally, two cases (2/33, 6.1%) died from unrelated causes.

The results of Log-rank analyses of the clinical parameters are summarized in **Table 2**. The survival analysis found that tumor subtype (χ2 = 16.118, *p*< 0.05), necrosis (χ2 = 6.174, *p*< 0.05) and surgical resection (complete resection vs. marginal resection; χ2 = 4.156, *p* < 0.05) were associated with overall survival (OS). DFS (disease-free survival) was related to tumor subtype (χ2 = 9.526, *p* < 0.05) and surgical resection (complete resection vs. marginal resection; χ2 = 7.605, *p* < 0.05). No significant difference was observed between the other survival factors with OS and DFS, including sex, age and tumor size.

**Table 2:** Survival data of the primary intrathoracic liposarcoma patients of the study.

Parameter	N (%)	Disease−free survival		Overall survival	
		Log−rank	*P*−value	Log−rank	*P*−value	
Gender	33	0.891	0.345	0.267	0.605	
Male	19 (57.6)					
Female	14 (42.4)					
Age	33	0.723	0.395	0.017	0.896	
<=50y	16 (48.5)					
>50 y	17 (51.5)					
Size	29	0.110	0.740	2.321	0.128	
<=10cm	7 (24.1)					
>10 cm	22 (75.9)					
Location	33	1.488	0.475	5.225	0.073	
Mediastinum	18 (54.6)					
Pleura space	14 (42.4)					
Lung	1 (3.0)					
Subtype	33	9.526	<0.05	16.118	<0.001	
Well-differentiated	19 (57.6)					
Dedifferentiated	12 (36.4)					
Myxoid pleomorphic	2 (6.0)					
Necrosis	33	2.795	0.095	6.174	<0.05	
Yes	8 (24.2)					
No	25 (75.8)					
Surgical resection	33	7.605	<0.05	4.156	<0.05	
Complete resection	14(42.4)					
Marginal resection	19(57.6)					

## Discussion

This study enrolled 43 primary intrathoracic liposarcomas, aged from 24 to 73 years (median, 53 years) with male predilection (M: F= 1.68:1). We reviewed previously reported primary intrathoracic liposarcoma (1990-2021) with available clinicopathological information in English literature(n=300), summarizing the series cases in **Table 3** and the rest in **Supplementary Table 1** (7–13). The majority of the historical cases were also older adults (median, 54 years) with a male predication (M: F= 1.46:1).

**Table 3:** Clinicopathologic features of previously reported primary mediastinal and intrathoracic liposarcomas of large series studies.

Published time	Case No.	Author	Gender (M:F)	Age (year)	Clinical Presentation	Size (cm)	Location	Histology	Molecular/IHC	Therapy	Follow-upInformation	Outcome
1964	8	Cicciarelli, F. E.,et al (7)	3:5	50 (13-70)	pain, cough, dyspnea, loss of weight	17.8 (14-22)	2 PM, 2 AM, 4 MC	8 Liposarcoma	NA	5 excision & RT, 2 excision,1 RT	6 recurrence	5 DOD, 3 AWD
1995	28	Klimstra DS, et al (8)	16:12	43 (14-72)	pain, cough, dyspnea,	15.7 (6- 40)	28 AM	15 WDL,7 ML,3 PL, 3 mixed-type	NA	28 excision	7 (7/22) recurrence	11 ANED, 4 AWD, 7 DOD,
2007	24	Hahn HP, et al. (9)	13:11	58 (3-72)	Dyspnea and cough	16 (2.2- 61)	9 AM,7 PM, 1 SM,7 mediastinum	10 WDL,8 DDL,2 ML,4 PL	NA	14 complete excision, 1 marginal excision +CT, 1 RT+CT	5 (5/15) recurrence 2 (2/15) metastasis	11 ANED, 1 AWD, 2 DOD, 1 DFU
2012	24	Boland JM, et al. (10)	13:11	53 (15-73)	NA	16 (8-30)	6 AM, 6PM, 2SM, 3 MM, 5MC, 1PS, 1AM/SM	8 WDL,6 DDL,2 ML, 4PL,3M-PL,1 Unclassifiable type	WDL:1/1*CPM*-(FISH), DDL:3/3 *CPM*+(FISH), ML:1/1*CPM*-, *DDIT3*-(FISH), PL:2/2 *CPM*-(FISH); MPL: 1/1 *DDIT3*-,1/1 *DDIT3*-, *EWS*-(FISH) Unclssifiable:1/1 *CPM*-, *FUS*- *DDIT3*-(FISH),	22 excision	8 (8/19) recurrence 5 (5/19) metastasis	6 ANED, 3 AWD, 9 DOD 3 Alive
2014	23	Chen M, et al. (11)	12:11	49 (16-72)	Chest pain, cough, dyspnea, shortness of breath	8 (4-39)	10mediastinum,9 PS, 4 lung	8 WDL, 4 DDL, 8 ML,3 PL	WDL:6/8 MDM2+, 6/8 CDK4+, 8/8 S100+(IHC) DDL: 3/4 MDM2+, 3/4 CDK4+, 4/4 S100+(IHC) ML:8/8 S100+(IHC)	17 complete excision,6 marginal excision	9(9/17) recurrence 6 (6/23) metastasis	10 DOD
2015	18	Ortega P, et al. (12)	11:7	57 (29-87)	Cough, dysphagia, and chest pain	15 (6-30)	18 PM	10 WDL,3 DDL, 3 ML,2 PL	WDL: 1/2 S100 +, 5/5 MDM2+(IHC); 2/2 *MDM2* +(FISH) DDL: 3/3 MDM2+, 0/3 S100+(IHC) ML: 3/3 S100+, 0/3 MDM2+(IHC)	12 complete excision, 4 marginal excesion+RT,1 RT	3 (3/13) recurrence 3 (3/13) metastasis	7 ANED, 3 AWD, 2 DOD, 1DFU
2019	31	Fu Z et al. (13)	19:12	45 (20- 64)	Chest tightness	10 (1.8-32)	16 AM, 8PM, 5 PS, 2 lung	6 WDL,3 DDL,13 ML,4PL,5Mixed-type	NA	17excision,8 excision+RT,3 RT, 1 excision +CT, 2excision+RT +CT	20 (20/31) recurrence 11 (11/31) metastasis	18 DOD

M, male; F, female; NA, not available; AM, anterior mediastinum; PM, posterior mediastinum; SM, superior mediastinum; MM, middle mediastinum; MC, multiple compartments; PS, pleural space; WDL, well-differentiated liposarcoma; DDL, dedifferentiated liposarcoma; ML, myxoid liposarcoma; PL, pleomorphic liposarcoma; M-PL, myxoid pleomorphic liposarcoma; IHC, immunohistochemistry; “+” positive, “-” negative; FISH, fluorescence *in situ* hybridization; RT, radiotherapy; CT, chemotherapy, ANED, alive with no evidence of disease; AWD, alive with disease; DFU, died from unrelated reasons; DOD, died of disease.

In this cohort, a preference for the mediastinum (23/43, 53.5%) was observed, which is in agreement with the tendency of previous cases (222/300, 74.0%). The percentage of our series (53.5%) was lower than that of previous studies (74.0%), which may be caused by our research analyzing all intrathoracic liposarcomas, whereas some large series studies focused on mediastinal tumors only (8, 9, 12). Only 4 cases in our cohort were located within the lung parenchyma (4/43, 9.3%), and 18 pulmonary liposarcomas were reported previously (18/300, 6.0%) (11, 13, 18–29). These results indicated that primary pulmonary liposarcoma is exceedingly rare but does exist.

According to the results of this study, the most significant difference between our series and previous studies is the distribution spectrum of the subtype. In our study, WDL/DDL was the overwhelming subtype (40/43, 93.0%), followed by MPL (2/43, 4.7%) and PL (1/43, 2.3%). Notably, ML was not identified in our study. The distribution spectrum differed significantly from the overall distribution of liposarcoma subtypes, in which WDL/DDL, ML and PL accounted for approximately 65%, 30%, and 5% of cases, respectively, and MPL was exceptionally rare. Moreover, our result is also different from to that of historical intrathoracic cases, in which WDL/DDL, ML, PL and MPL accounted for 56.7% (170/300), 21.3% (64/300), 11.3% (34/300) and 2.7% (8/300), respectively. Notably, the subtype distribution of our thoracic liposarcoma is similar to that of primary retroperitoneal liposarcoma, in which WDL/DDL is the predominant subtype and ML and PL are vanishingly rare (30).

The 21 WDLs in our study comprised lipoma-like (61.9%), inflammatory (9.5%), and mixed subtype tumors (28.6%). Most historical WDLs with available subtype information (n=54), were also lipoma-like (12/54, 22.2%) and mixed-subtype (32/54, 59.3%). Conspicuous myxoid change was observed in 6 of our cases (28.6%) and appeared to be more common than in reported tumors with available descriptions (12/66, 18.2%) (9, 10, 12). Attention should be given to these cases, as some WDLs can show extensive myxoid changes mimicking ML.

It should be mentioned that only 8 historical WDLs (6.8%) were analyzed by FISH, including 6 *MDM2*-amplified cases, 1 case with equivocal *MDM2*-FISH results, and 1 *CPM*-nonamplified case (10, 12, 20, 31–34). In our study, all 18 tested WDLs were 12q13-15 amplified, including 17 *MDM2*-amplified cases and 1 *FRS2-*amplified/*MDM2-*nonamplified/*CDK4-*nonamplified tumor (case 4). We reported the first *FRS2*+/*MDM2*-/*CDK4*- WDL in English literature. The position of the *FRS2* gene is close to the *MDM2* and *CDK4* genes within the 12q13-15 chromosomal region. In 2011, Wang et al. identified consistent amplification of the *FRS2* gene in 57 WDL/DDLs (100%) (35).Subsequently, our research found a high amplification frequency of the *FRS2* gene in WDL/DDLs (136/146, 93.2%) and low-grade osteosarcoma (21/22, 95%), slightly lower than that of *MDM2* (100%). These results indicated that FISH analysis of the *FRS2* gene could also be a useful ancillary tool for the diagnosis of WDL/DDL (17, 36). Moreover, in addition to *MDM2* and *CDK4*, aberrations of other genes at 12q13-15 may also participate in the pathogenesis of this entity, and comprehensive molecular analysis in challenging cases is valuable.

In the 19 DDLs, 13 tumors (68.4%) exhibited a conventional dedifferentiation pattern, and the other 6 cases showed uncommon morphology, including an IMT-like pattern (n=3, 15.8%), low-grade dedifferentiation (n=2, 10.5%) and DDL with leiomyosarcomatous differentiation (n=1, 5.3%). It should be mentioned that IMT-like features are a recently described histologic pattern of DDL, while none of the intrathoracic cases have been described in the English literature (37–39). In the 52 reported DDLs, 10 of them (10/52, 19.2%) exhibited low-grade dedifferentiation (9, 10, 12, 40–42). Notably, 1 case was DDL with leiomyosarcomatous differentiation, mimicking smooth muscle tumor and pulmonary adenofibroma histologically. However, typical areas of WDL were found after extensive sampling. Furthermore, the identification of *MDM2* amplification helped the final diagnosis. To the best of our knowledge, only 1 primary intrathoracic DDL with leiomyosarcomatous differentiation has been described (12). In the study cohort, all tested DDL tumors (16/16, 100%) exhibited *MDM2* amplification. However, only 11 of 52 (21.1%) historical cases were subjected to FISH analysis, and all of them displayed positive results (10, 18, 40, 42–47). We endorsed ancillary tests for cases in rare locations, such as the mediastinum, lung and/or ambiguous morphology.

Two MPLs were identified in this study. As an exceptionally rare subtype of liposarcoma, only 38 MPLs have been reported previously. Of the 37 reported cases with available information, MPLs have a predilection for the mediastinum (15/37, 40.6%), suggesting although MPL is an extremely rare subtype of liposarcoma, it is not the rarest liposarcoma variant in the mediastinum (10, 15, 48–55). The 2 MPL patients in our study were 24 and 49 years old. The median age of cases from case reports and series study was 17 years and 35 years, respectively (55). These result suggested MPL is more prone to occur in young age group.

The MPL tumors of this cohort simultaneously harbored pleomorphic liposarcoma-like areas and myxoid liposarcoma-like areas, with a plexiform vasculature pattern in a myxoid background, similar to previously reported cases. The MPLs in our study and historically tested MPL cases were all negative for *MDM2* amplification or *DDIT3* rearrangement, indicating the phenotype of MPL was different from that of WDL/DDL or ML (10, 48, 50–54). The WES results of our MPL case showed numerous chromosome gain and loss loci, similar to 2 previous aCGH studies of 2 MPLs, and 1 large series study of MPLs (50, 51, 55). Moreover, our results found losses are more frequent than gains in MPL case, which were in agreement with Creytens et al. but different from other studies, and more MPL cases are needed to verify the finding. Furthermore, both our research and previous studies found complex chromosomal aberrations in MPL. However, these results revealed MPL showed a simpler pattern of chromosome alterations than conventional PL, with focal copy number changes rather than whole chromosomal gains and losses.

MPL may be related to Li-Fraumeni syndrome (LFS) associated with germline *TP53* mutations. Both MPL tumors of our study harbored somatic *TP53* mutations, without association with LFS. Including our 2 cases, *TP53* mutation was found in 78% of MPLs (7/9) (15, 51–54). These findings reminded us *TP53* mutation might play a role in the pathogenesis of MPL. *RB1* gene deletion was found in 10 of 15 (66.7%) historical MPLs and 1 MPL was reported to have *KMT2D* gene mutation, while our 2 MPL cases were negative for such genetic changes (15, 51, 55). It should be pointed out that conventional PL also harbors frequent *TP53* and *RB1* mutations (56), implying that aberrant genes of conventional PL overlapped with those of MPL. Further study is needed to identify the relationship and difference between these two entities.

Only 1 conventional PL was found in our study with the identification of typical of bizarre, giant lipoblasts and without amplification of *MDM2*/*CDK4*/*FRS2* genes. In previous studies, PL was also rarely seen in this location and accounted for 11.3% (34/300) of reported cases. The majority of historical PLs were diagnosed based on morphology only, and only 2 cases were found to be *CPM* nonamplified (10). Therefore, PL in the thorax is scarce and the diagnosis of PL in this location is challenging. Molecular analysis is needed to rule out the possibility of other subtypes of liposarcoma.

Primary intrathoracic ML was not identified in our cohort. In fact, 2 cases were coded as intrathoracic MLs at the beginning. However, one was proved to be a metastatic tumor, and another was revised as DDL (case 32) which was *MDM2*-amplified but *DDIT3*-nonrearranged. Although 64 primary intrathoracic MLs have been reported, only one tumor was *DDIT3*-rearranged (10, 26). Hence, primary intrathoracic ML does exist, but it may be quite rare.

The correct diagnosis and classification of intrathoracic liposarcoma subtypes is of considerable importance and may be challenging for difficult cases, particularly in small biopsy specimens. Moreover, intrathoracic liposarcomas should be differentiated from a variety of other types of neoplastic and nonneoplastic lesions, such as benign adipose tissue tumors, inflammatory lesions, and other spindle cell lesions. It should be emphasized that intrathoracic DDL especially in the pleuropulmonary area is extremely rare and can mimic other types of adipocytic and nonadipocytic tumors, such as IMT, solitary fibrous tumor (SFT), synovial sarcoma, malignant peripheral nerve sheath tumor (MPNST), smooth muscle tumor, pulmonary adenofibroma, intimal sarcoma, sarcomatoid carcinoma, and other types of liposarcoma.

In this study, one lipoma-like WDL (case 13) and one inflammatory WDL case (case 16) were diagnosed as lipoma and inflammatory pseudotumor, respectively, at a local hospital. Careful histological inspection can aid in identifying atypical adipocytes of varying sizes, especially bizarre, hyperchromatic stromal cells. More importantly, MDM2 and CDK4 nuclear positivity, especially *MDM2* gene amplification, can be invaluable in distinguishing WDL from lipoma and inflammatory lesions.

IMT is the more common tumor type in the pleuropulmonary area, outnumbering liposarcomas. This study cohort comprised 3 IMT-like DDL tumors and could be easily confused with IMT. However, extensive sampling identified typical areas of a WDL component, suggestive of the diagnosis of DDL. More importantly, IMT can be excluded because of the presence of high-level amplification of the *MDM2* locus and the absence of *ALK, ROS1*, *NTRK3, RET*, or *PDGFRB* gene rearrangement. It should be pointed out that the diagnosis of *MDM2*-amplified IMT should be extremely cautious, as *MDM2* amplification is the genetic hallmark of DDL, although a few IMT cases harboring *MDM2* amplification have been reported (57, 58).

SFT is one of the commonest pleuropulmonary soft tissue tumors and might share some morphologic features with DDL. SFT could be distinguished from DDL in the following aspects. First, SFT usually exhibits strong and diffuse nuclear positivity of STAT6 although a subset of DDL may also show moderate or weak STAT6 expression caused by the *STAT6* amplification (59). Most importantly, SFT can be excluded because of the presence of *MDM2* gene amplification and absence of *NAB2*-*STAT6* gene fusion (15, 60).

Primary pleuropulmonary synovial sarcoma has gradually been recognized as a clinicopathological entity. Sometimes the histologic features of DDL and synovial sarcoma can overlap significantly, especially in small biopsy samples. Careful morphologic inspection and ancillary immunohistochemical markers including EMA, TLE1, cytokeratins, and MDM2 are helpful in distinguishing between these lesions. Of note, ancillary molecular studies testing of t(X;18)(p11.2;q11.2) for synovial sarcoma and *MDM2* amplification can be invaluable in secure the diagnosis (61).

MPNST could resemble DDL and appears to be the most challenging tumor type in the differential diagnosis of DDL in any location. MPNST usually shows complete loss of staining for H3K27me3 but negativity for MDM2 expression. Notably, detection of *MDM2* amplification, combined with the absence of the genetic loss of *SUZ12* or *EED*, can help to confirm the diagnosis of DDL (62).

This cohort comprised one peculiar DDL with leiomyosarcomatous differentiation (case no. 33), posing diagnostic challenges on morphologic grounds only. The distinction can be aided through extensive sampling of the lesion. For example, in the current case, 10 blocks were taken at the very beginning, and the tumor was almost entirely composed of smooth muscle tumor elements, mimicking smooth muscle tumor. Additionally, the tumor component within the lung parenchyma showed a leaf-like pattern and could be confused with pulmonary adenofibroma. However, typical areas of WDL were found both in the pleural areas and pulmonary areas of the mass after an extra 12 blocks were taken. Moreover, the identification of *MDM2* amplification in the tumor further helped us to make the diagnosis.

Four DDLs of our study were located within the lung parenchyma, which were extremely rare in this site. It should be pointed out that all the 4 DDLs were *MDM2*-amplified. Moreover, the imaging of the 4 patients did not found tumors elsewhere of the body. The WDL component was identified in 1 case and lipomatous components were found radiologically in 2 biopsy samples. While the WDL component was not observed in another surgical resected tumor (case 34). Such case might be confused with tumors carrying *MDM2* amplification, such as intimal sarcoma and a few pulmonary sarcomatoid carcinomas (PSC). Firstly, the diagnosis of intimal sarcoma was excluded as the different location of the two entities. Intimal sarcomas mainly arise from pulmonary arteries and major systemic arteries, while the DDLs were all located within the lung parenchyma (63). Secondly, all the four documented *MDM2*-amplified PSCs had adenocarcinoma components. In contrast, carcinomatous component was not detected in our DDL case (64, 65). In combination with the clinicopathologic, genetic and radiologic results, this case (case 34) was diagnosed as DDL.

In fact, both of the MPL cases in our series were diagnosed as PL in the local hospital. MPL is an exceptionally rare emerging entity of liposarcoma, and most general surgical pathologists are not familiar with this peculiar tumor. However, careful histological inspection revealed that both tumors exhibited conspicuous myxoid areas, showing mixed features of classic PL and ML. Additionally, these tumors were negative for *DDIT3* rearrangement and *MDM2* amplification. Finally, the diagnosis of MPL was established.

PL is extremely rare in the intrathoracic location and should be distinguished from other tumors especially DDL with homologous lipoblastic differentiation. It is noteworthy to point out that a minority of DDL cases can exhibit pleomorphic liposarcoma-like differentiation, making it indistinguishable from PL (66, 67). DDL usually comprises typical areas of WDL component within the tumor. Importantly, negativity for MDM2 overexpression, especially lack of amplification of *MDM2* gene can help distinguish PL from the above mentioned variant of DDL.

The results of this study showed that tumor subtype was an important prognostic factor for the OS of intrathoracic liposarcoma patients. In our cohort, only 3 of 19 (15.8%) WDL and 3 of 12 (25.0%) DDL cases, but both MPLs (2/2,100%), died of disease. In previously reported cases with follow-up information, disease-related death was found in 5 of 73 (6.8%) WDLs, 10 of 42 (23.8%) DDLs and 15 of 26 (57.7%) MPL cases. Similar to our study, the mortality was highest in MPL and lowest in WDL cases, suggesting that the intrathoracic tumor subtype was related to the overall survival of the cases. Our research also found that OS and DFS were related to marginal or complete resection of the tumor, as none of the 13 patients who received complete tumor resection died of disease, while 44.5% (8/18) patients who underwent marginal resection died of disease. Chen et al. found that surgical resection was associated with the OS of tumors, further indicating that the surgical procedure can influence the behavior and prognosis of the disease (11).

In conclusion, we present clinicopathological and molecular features of 43 primary intrathoracic liposarcomas. In our study, WDL/DDL is the overwhelming subtype, followed by MPL and PL. Notably, ML was not identified. MPL is extremely rare in liposarcoma, but it is not the rarest subtype in thorax. One *FRS2*+/*MDM2*-/*CDK4*- WDL was identified, indicating that analysis of the *FRS2*, in combination with *MDM2* and other genes located at 12q13-15, may more precisely characterize WDL/DDLs. Both MPLs exhibited somatic *TP53* mutations, showing overlapping features with conventional PL. MPL is the most fatal subtype of this site, suggesting that correct classification is of considerable significance.

## Data availability statement

The data presented in the study are deposited in the NCBI database, accession number SAMD00514799 and SAMD00514800.

## Author contributions

YX: data analysis, histopathological examinations and writing. WJ: histopathological examinations and writing. WZ: data analysis and histopathological examinations. RP: data analysis. MC: help molecular experiments. TL: data analysis. HP: help molecular experiments. XH: histopathological examinations. HC: histopathological examinations. ZZ: histopathological examinations. HZ: the corresponding author, study design, histopathological and molecular examinations, and the manuscript revision. All authors contributed to the article and approved the submitted version.

## Funding

This work was supported by the National Natural Science Foundation of China (No. 81972520) and the 1·3·5 project for disciplines of excellence–Clinical Research Incubation Project, West China Hospital, Sichuan University (No. 2018HXFH011).

## Conflict of interest

The authors declare that the research was conducted in the absence of any commercial or financial relationships that could be construed as a potential conflict of interest.

## Publisher’s note

All claims expressed in this article are solely those of the authors and do not necessarily represent those of their affiliated organizations, or those of the publisher, the editors and the reviewers. Any product that may be evaluated in this article, or claim that may be made by its manufacturer, is not guaranteed or endorsed by the publisher.
