# Supplementary material for: Primary intrathoracic liposarcomas: A clinicopathologic and molecular study of 43 cases in one of the largest medical centers of China
Source: Front Oncol. 2022 Aug 17;12:949962. doi: 10.3389/fonc.2022.949962 (PMC9432863; doi:10.3389/fonc.2022.949962)
Supplement: Supplementary file 1 [file Table_1.docx]

SUPPLEMENTARY TABLE 1. Clinicopathologic Features of 152 Primary Intrathoracic Liposarcomas in Historical Case Reports

| Case No. | references | Age  /sex | Symptoms | Size (cm) | Location | Histology | | IHC results | | Genetic results | | Treatment | | Outcome/Follow-up duration | |  |
| --- | --- | --- | --- | --- | --- | --- | --- | --- | --- | --- | --- | --- | --- | --- | --- | --- |
| 1 | Suzuki, T. et al^1^ | 57/M | NA | 23 | Posterior mediastinum | | WDL | | NA | | ND | | Complete resection | | NED/26 mo | |
| 2 | Alhames, S. et al^2^ | 46/M | Fatigue, shortness of breath and chest pain | 35 | Anterior mediastinum | | WDL | | NA | | ND | | Complete resection | | NED/12 mo | |
| 3 | Gaikwad, N. M. et al^3^ | 53/F | Cough and dyspnea | 20 | Anterior mediastinum | | WDL | | NA | | ND | | Complete resection | | NED/24 mo | |
| 4 | Wang, L. et al^4^ | 56/F | Chest pain | 6.1 | Descending aorta | | PL | | MDM2+ | | ND | | ND | | DOD/1 mo | |
| 5 | Furlan, K. et al^5^ | 33/F | Chronic cough | 10.8 | Anterior and superior mediastinum | | WDL | | MDM2+, CDK4+, P16+ | | *MDM2*:D12Z3 =1.89(FISH) | | Complete resection | | NED/12 mo | |
| 6 | Iwamoto, N. et al^6^ | 77/M | Cough | 11.9 | Anterior mediastinum | | WDL (sclerotic) | | CD34+, MDM2+, STAT6- | | *MDM2* +(FISH) | | Complete resection | | NED/60 mo | |
| 7 | Kang, L. H. et al^7^ | 66/M | NA | 4.5 | Pleura | | WDL | | MDM2+, CDK4+ | | ND | | Complete resection | | NA | |
| 8 | Ye, Y. W. et al^8^ | 38/F | Dysphagia and dyspnea | NA | Posterior mediastinum | | DDL | | Desmin +, CD34+, S100+ | | *MDM2* +(FISH) | | Complete resection | | NED/20 mo | |
| 9 | Chen, H. G. et al^9^ | 75/M | Dysphagia | 10.5 | Posterior-inferior mediastinum | | DDL | | Vimentin+, S100+, CD34 +, CD68+, Desmin +, SMA- | | ND | | Complete resection | | Recurrence at 14,29 mo;DOD/ 54 mo | |
| 10 | Mei, X. et al^10^ | 54/M | Chest pain | 30 | Mediastinum | | Lipo-LMS | | S100+, SMA +, Deamin +, Calesmon + | | ND | | Complete resection | | DOD/36 mo | |
| 11 | Boatright, C. et al^11^ | 54/M | NA | 4.6 | Anterior mediastinum | | DDL (low-grade) | | NA | | *MDM2* +(FISH) | | Complete resection | | NA | |
| 12 | Prabhakar, Nidhi et al^12^ | 32/M | Chest pain and  shortness of breath | 20.8 | Right hemithorax | | ML | | S100+ | | ND | | chemotherapy and  radiotherapy | | AWD/NA | |
| 13 | Zhang, M. et al^13^ | 30/M | Dysphagia | 30 | Anterior superior mediastinum | | WDL | | NA | | *MDM2* +(FISH) | | Complete resection | | NED/8 mo | |
| 14 | Ishtiaq, R. et al^14^ | 51/M | NA | 18.4 | Thorax | | ML | | NA | | NA | | Complete resection | | NA | |
| 15 | Ashraf, U. et al^15^ | 76/M | Chest pain | 10 | Left hemithorax | | DDL | | NA | | NA | | Complete resection | | AWD/7 mo | |
| 16 | Matsukuma, S. et al^16^ | 45/M | Chest pain | 10 | Left pleural | | DDL | | MDM2+ | | NA | | Incomplete resection | | DOD/4 mo | |
| 17 | Kandakure, P. R. et al^17^ | 50/F | Cough and dyspnea | 45 | Posterior mediastinum | | Liposarcoma | | NA | | NA | | NA | | NA | |
| 18 | Yang, Y. S. et al^18^ | 63/M | Cough and dyspnea | 20 | Anterior mediastinum | | WDL | | vimentin+, CD 34+ | | NA | | Complete resection | | NED/24 mo | |
| 19 | Soeroso, N. N. et al^19^ | 54/M | Cough and dyspnea | 50 | Anterior mediastinum | | DDL | | S100+, vimentin+, | | NA | | Incomplete resection | | Recurrence at 6 mo, CT; AWD/7 mo | |
| 20 | Chen, C. et al^20^ | 43/M | Dyspnea | 28 | Both chest cavities | | WDL | | NA | | NA | | Complete resection | | NED/40 mo | |
| 21 | Kim, S. Y. et al ^21^ | 51/F | Cough and dyspnea | 9.1 | Anterior mediastinum | | DDL | | NA | | *MDM2* +(FISH) | | Complete resection | | NED/12 mo | |
| 22 | Pui, W. C. et al^22^ | 51/M | Cough | NA | Left thorax | | ML | | NA | | NA | | Complete resection | | NED/24 mo | |
| 23 | Nguyen, D. C. et al^23^ | 48/F | Cough and dyspnea | 16 | Anterior mediastinum | | WDL | | NA | | NA | | Complete resection | | NED/6 mo | |
| 24 | Pusiol, T. et al^24^ | 46/M | Chest pain | NA | Left hemithorax | | ML | | NA | | NA | | NA | | NA | |
| 25 | Takenaka, Masaru et al^25^ | 73/M | NA | 17 | Mediastinum | | WDL | | NA | | NA | | Complete resection | | Recurrence at 71 mo, resection again; NED/36 mo | |
| 26 | Tong, J. J. et al^26^ | 42/F | Dyspnea | 30 | Posterior mediastinum | | Lipo-LMS | | CDK4+, P16 +, SMA + | | *MDM2* +(FISH) | | Complete resection | | NED/14 mo | |
| 27 | Galli, A. et al^27^ | 55/M | Laterocervical lump | 15 | Anterior mediastinum | | WDL (mixed-subtype) | | MDM2+ | | NA | | Complete resection+ RT | | NED/38 mo | |
| 28 | Weaver, H. L. et al^28^ | 72/M | NA | 30.5 | Mediastinum | | DDL | | NA | | NA | | Complete resection | | NED/4 mo | |
| 29 | Sugiura, Y. et al^29^ | 50/M | Dyspnea | NA | Anterior mediastinum | | WDL | | NA | | NA | | Complete resection | | NED/36 mo | |
| 30 | Edagawa, M. et al^30^ | 64/F | NA | 9.2 | Middle mediastinum | | WDL (inflammatory) | | CDK4+, MDM2 +, p16 + | | NA | | Complete resection | | NED/8 mo | |
| 31 | Khan, M. H. et al^31^ | 43/M | Chest pain | NA | Anterior mediastinum | | ML | | NA | | NA | | Complete resection+ RT | | NED/10 mo | |
| 32 | Wang, F. et al ^32^ | 43/F | NA | 21 | Left thorax | | ML | | S100 + | | NA | | Complete resection | | NED/8 mo | |
| 33 | Toda, M. et al^33^ | 64/M | Dyspnea | 36.5 | Anterior mediastinum | | DDL | | NA | | NA | | Complete resection | | NED/20 mo | |
| 34 | Huang, W. et al^34^ | 57/M | Dyspnea | 27 | Anterior mediastinum | | WDL | | NA | | NA | | Complete resection | | NA | |
| 35 | Zhao, C. et al^35^ | 63/M | Dyspnea, Chest pain | 24 | Posterior mediastinum | | liposarcoma | | NA | | NA | | Complete resection | | Recurrence at 12mo and 24 mo, resection/ DOD, 40 mo | |
| 36 | Lin, F. et al^36^ | 47/M | Cough and dyspnea | 30 | Posterior mediastinum | | WDL | | NA | | NA | | Complete resection | | NED/25 mo | |
| 37 | Arava, S. et al^37^ | 77/M | Cough and dyspnea | 14 | Anterior mediastinum | | WDL (sclerotic) | | S100 +, SMA-, Desmin- | | NA | | RT+CT | | NA | |
| 38 | Li, Y. Z. et al^38^ | 74/F | Asymptomatic |  | pleural and Posterior mediastinum | | WDL | | NA | | NA | | Complete resection | | Recurrence at 60 mo, resection/ NED, 60 mo | |
| 39 | Biswas, A. et al^39^ | 52/F | Chest pain | 22 | Right thorax | | Liposarcoma | | S100+, CD34+ | | *MDM2*-, *DDIT3*- | | Complete resection | | NED/24 mo | |
| 40 | Hamanaka, K. et al^40^ | 74/M | Cough | 11.4 | left middle and posterior mediastinum | | DDL | | MDM2 +, CDK4+ | | NA | | Complete resection | | NED/8 mo | |
| 41 | Mani, V. R. et al^41^ | 28/F | Asymptomatic | 28 | Anterior mediastinum | | WDL (sclerotic) | | S100+, CD34+, MDM2 + | | NA | | Complete resection | | NED/ NA | |
| 42 | Harth, S. et al ^42^ | 83/M | Cough and dyspnea | 24.2 | Anterior mediastinum | | DDL | | ND | | *MDM2* +, *CDK4*+(FISH) | | Complete resection | | DOD/ 6 mo | |
| 43 | Ma, J. et al^43^ | 27/M | Dyspnea | 23 | Posterior mediastinum | | WDL | | CDK4+ | | NA | | Complete resection+RT | | NED/2 mo | |
| 44 | Kawamura, T. et al^44^ | 61/M | Aphagia |  | Anterior mediastinum | | WDL | | NA | | NA | | RT | | Died of bleeding | |
| 45 | Arraras-Martinez, M. J. et al^45^ | 68/M | Dyspnea | 17.5 | Posterior mediastinum | | liposarcoma | | Vimentin+, SMA + | | NA | | Incomplete resection+CT | | Recurrence at 12 mo, CT/ DOD, 15 mo | |
| 46 | Rena, O. et al^46^ | 58/M | Dyspnea | 18 | Mediastinum | | WDL | | NA | | NA | | Complete resection | | NED/6 mo | |
| 47 | Fujimoto, R. et al^47^ | 64/F | Asymptomatic | NA | Anterior mediastinum | | DDL (osteosarcoma-like differentiation) | | NA | | NA | | Complete resection | | Recurrence at 24mo, 108mo, resection/ NED, 108 mo | |
| 48 | Asaka, S. et al^48^ | 45/F | Asymptomatic | 12.7 | Mediastinum | | DDL | | MDM2 +, CDK4+ | | *MDM2* +, *CDK4*+(FISH) | | Complete resection | | NED/11 mo | |
| 49 | Carrillo, B. Ja et al^49^ | 49/F | Dyspnea | 19 | Left thorax | | PL | | NA | | NA | | Incomplete resection | | AWD/6 mo | |
| 50 | Chen, G. et al^50^ | 49/M | Dyspnea | 10 | Superior mediastinum | | PL | | NA | | NA | | Complete resection+ RT | | NED/14 mo | |
| 51 | Longano, A. et al^51^ | 74/M | Cough |  | Lung | | DDL (rhabdomyoblastic and chrondroblastic differentiation) | | S100+, desmin +, actin+, and myogenin + | | *MDM2* +(FISH) | | Complete resection+ RT | | NED/12 mo | |
| 52 | Fukuhara, S. et al^52^ | 77/M | Dyspnea | 36 | Anterior mediastinum | | WDL | | NA | | NA | | Complete resection | | NED/24 mo | |
| 53 | Hirano, Y. et al^53^ | 53/F | Chest discomfort | 31 | Anterior mediastinum | | WDL | | NA | | NA | | Complete resection | | NED/7 mo | |
| 54 | Billè, Andrea et al^54^ | 29/M | Dyspnea | 27 | Posterior mediastinum and thorax | | WDL | | MDM2 +, S100+ | | NA | | Complete resection | | Recurrence at 30 mo, CT/ AWD, 72 mo | |
| 55 | Zhou, L. F. et al^55^ | 38/F | Dyspnea | NA | Right thorax | | WDL | | NA | | *PIK3CA* mutation (PCR) | | NA | | NA | |
| 56 | Kashu, Y. et al^56^ | 63/M | Dyspnea | 31 | Left thorax | | WDL | | NA | | NA | | Complete resection | | NED/22 mo | |
| 57 | Matsuo, T. et al^57^ | 52/M | Dyspnea | NA | Right thorax | | DDL | | NA | | NA | | Incomplete resection+ RT | | AWD/48 mo | |
| 58 | Decker, J. R. et al^58^ | 74/F | Dyspnea | 18 | Right thorax | | WDL | | NA | | NA | | Complete resection | | NED/32 mo | |
| 59 | Elsayed, H. et al^59^ | 47/M | Dyspnea | 18 | Right thorax | | WDL (sclerotic) | | NA | | NA | | Complete resection | | NA | |
| 60 | Shoji, F. et al^60^ | 45/F | Cough | NA | Posterior mediastinum | | Liposarcoma | | NA | | NA | | Incomplete resection+ RT | | AWD/26 mo | |
| 61 | Sivaraman, A. et al^61^ | 55/M | Chest pain | 30 | Anterior mediastinum | | PL | | NA | | NA | | Complete resection +CT | | NED/3 mo | |
| 62 | Wiedemann, D. et al^62^ | 64/M | Dyspnea | NA | Mediastinum | | WDL | | NA | | NA | | Complete resection | | NED/12 mo | |
| 63 | Taki, K. et al^63^ | 39/M | Chest pain | 40 | Posterior mediastinum | | WDL | | NA | | NA | | Complete resection | | NED/14 mo | |
| 64 | Okuno, M. et al ^64^ | 39/F | Chest discomfort | 25 | Anterior mediastinum | | WDL | | NA | | NA | | Complete resection | | NED/31 mo | |
| 65 | Lin, Y. Y. et al^65^ | 46/F | Laterocervical lump | NA | Anterior mediastinum | | WDL | | NA | | NA | | Complete resection | | NED/8 mo | |
| 66 | Saeed, M. et al^66^ | 17/F | Dyspnea | 22 | Anterior mediastinum | | PL | | CDK4- | | NA | | Complete resection +CT | | NA | |
| 67 | Liu, L. G. et al^67^ | 44/M | Chest pain | 30 | Posterior mediastinum | | ML | | S100+, CD34+ | | NA | | Complete resection | | NED/4 mo | |
| 68 | Dagli, A. F. et al^68^ | 56/F | Chest pain | NA | Left thorax | | ML | | NA | | NA | | NA | | NA | |
| 69 | Anand Rajan, K. D. et al^69^ | 11/F | Cough, dyspnea | 31 | Posterior mediastinum | | Liposarcoma | | NA | | NA | | Complete resection | | NED | |
| 70 | Konno, S. et al^70^ | 73/M | Cough, dyspnea | 17 | Posterior mediastinum | | DDL | | NA | | NA | | RT+CT | | DOD/72 mo | |
| 71 | Gethin-Jones, T. L. et al^71^ | 70/M | Dyspnea | 11 | Mediastinum | | WDL | | NA | | NA | | Complete resection +RT | | NA | |
| 72 | Chaput-Dugas, M. E. et al^72^ | 45/F | Gastroesophageal reflux | 13 | Anterior mediastinum | | WDL | | S100+, CD34+ | |  | | Complete resection | | NED/24 mo | |
| 73 | Thomaz, F. B. et al^73^ | 50/M | Chest pain | 11.5 | Left thorax | | PL | | NA | | NA | | Complete resection | | NED/12 mo | |
| 74 | Gasiorowski, L.^74^ | 49/F | Cough | 31 | Left thorax | | Liposarcoma | | NA | | NA | | Complete resection | | NED/15 mo | |
| 75 | Coulibaly, B. et al^75^ | 34/F | Dyspnea | 20 | Mediastinum | | DDL | | MDM2 +, CDK4+ | | *MDM2* +, *CDK4*+(FISH) | | Complete resection, | | Recurrence at 15 and 96 month, RT+CT/DOD,99 mo | |
| 76 | Berry, M. F. et al^76^ | 38/M | Cough | 29.3 | Thorax | | ML | | NA | | NA | | Complete resection+CT | | NED/16 mo | |
| 77 | Fukai, R. et al^77^ | 56/M | Asymptomatic | 16.5 | Anterior mediastinum | | DDL (low-grade) | | NA | | NA | | Complete resection | | NED/36 mo | |
| 78 | Shoji, Tsuyoshi et al^78^ | 82/M | Chest discomfort | 16.3 | Superior mediastinum | | WDL | | NA | | NA | | Complete resection | | NED/10 mo | |
| 79 | Achir, A. et al^79^ | 49/M | Dyspnea | 12 | Lung | | PL | | NA | | NA | | Complete resection | | NED/36 mo | |
| 80 | Alloubi, I. et al^80^ | 58/M | Cough, dyspnea | NA | Left thorax | | ML | | NA | | NA | | Complete resection+RT | | NED/9 mo | |
| 81 | Greif, J. et al^81^ | 62/M | Dyspnea | 10 | Anterior mediastinum | | Liposarcoma | | NA | | NA | | Complete resection+RT | | Recurrence at 8 mo, CT/ DOD， 24mo | |
| 82 | Barbetakis, N. et al^82^ | 68/M | Chest pain, dyspnea | 9 | Left thorax | | WDL | | NA | | NA | | Complete resection | | NED/9 mo | |
| 83 | Benchetritt, M. et al^83^ | 76/M | Pneumonia | 20 | Posterior mediastinum, Left thorax | | DDL (low-grade) | |  | | *MDM2* +, *CDK4*+(FISH) | | Complete resection | | Died of heart infraction | |
| 84 | Goldsmith, P. et al^84^ | 42/M | Cough, dyspnea | NA | Left thorax | | ML | | NA | | NA | | Complete resection | | Recurrence at 6 mo, resection and CT/ AWD | |
| 85 | Goldsmith, P. et al^84^ | 80/F | Dyspnea | NA | Left thorax | | ML | | NA | | NA | | Complete resection | | DOD/8 mo | |
| 86 | Raghavan, R. et al^85^ | 38/M | Dyspnea | NA | Posterior mediastinum | | ML | | NA | | NA | | Complete resection | | NED/6 mo | |
| 87 | Munjal, K. et al^86^ | 40/F | Cough, dyspnea | 8.9 | Anterior mediastinum | | ML | | NA | | NA | | Complete resection | | NA | |
| 88 | Romero-Guadarrama, M. B. et a;^87^ | 66/M | Cough, dyspnea | NA | Anterior mediastinum | | PL | | NA | | NA | | NA | | NA | |
| 89 | Marulli, G. et al^88^ | 29/F | Cough, dyspnea | NA | Posterior mediastinum, Left thorax | | ML | | NA | | NA | | Complete resection | | Recurrence at 12 mo, resection / NED, 24mo | |
| 90 | Peng, C. et al^89^ | 56/F | Dyspnea | NA | Left thorax, mediastinum | | WDL | | NA | | NA | | Complete resection | | NED/18 mo | |
| 91 | Trahan, S. et al^90^ | 48/F | NA | 8 | Lung | | WDL | | NA | | *MDM2* +(FISH) | | Complete resection | | NA | |
| 92 | Marolla, A. et al^91^ | 73/F | NA | 2 | Posterior mediastinum | | Liposarcoma | | NA | | NA | | Complete resection | | NED/8 mo | |
| 93 | Hirai, S. et al^92^ | 64/M | Hoarseness | 6.4 | Anterior mediastinum | | DDL (undifferentiated pleomorphic sarcoma) | | NA | | NA | | Complete resection | | NED/14 mo | |
| 94 | Punpale, A. et al^93^ | 62/M | Dyspnea | NA | Posterior mediastinum | | WDL | | NA | | NA | | Complete resection | | NA | |
| 95 | Minniti, A. et al^94^ | 50/M | Chest pain | 13 | Posterior mediastinum | | WDL (Lipoma-like) | | NA | | NA | | Complete resection +RT | | NED/12 mo | |
| 96 | Ibe, T. et al^95^ | 36/M | Chest pain | 14 | Lung | | PL | | NA | | NA | | Complete resection | | DOD/ 2mo | |
| 97 | Loddenkemper, C. et al^96^ | 49/F | Cough, dyspnea | 9 | Lung | | DDL | | S100-, CD34- | | NA | | Complete resection | | NED/16 mo | |
| 98 | Takanami, I. et al^97^ | 59/M | Chest pain | 12 | Right thorax | | DDL (leiomyosarcoma and osteosarcoma-like) differentiation | | SMA+, desmin +,  HHF-35 +, CD34 + | | NA | | Complete resection | | NED/6 mo | |
| 99 | Ohta, Yasuhiko et al^98^ | 59/F | Dyspnea | 21 | Thorax | | WDL | | NA | | NA | | Complete resection | | NED/6 mo | |
| 100 | Noji, T. et al^99^ | 53/M | NA | 5 | Anterior mediastinum | | ML | | NA | | NA | | Complete resection | | Recurrence at 60 mo, resection/ NED, 75 mo | |
| 101 | Said, M. et al^100^ | 28/F | Chest pain | NA | Lung | | ML | | NA | | NA | | Resection | | Died at operation | |
| 102 | Kim, S. H. et al^101^ | 45/M | Dyspnea | 16 | Anterior mediastinum | | Liposarcoma | | NA | | NA | | Complete resection | | NED | |
| 103 | Paci, M. et al^102^ | 74/F | NA | NA | Posterior mediastinum | | WDL (lipoma-like) | | NA | | NA | | Complete resection | | NED/6 mo | |
| 104 | Mase, T. et al^103^ | 48/M | Dyspnea | 10.5 | Left thorax | | WDL (sclerotic) | | NA | | NA | | Complete resection | | NED/8 mo | |
| 105 | Sakamaki, Y. et al^104^ | 76/M | Asymptomatic | 12 | Posterior mediastinum | | WDL | | NA | | NA | | Complete resection | | NED/24 mo | |
| 106 | Kara, M. ^105^ | 52/F | Chest pain | 33 | Right thorax | | Liposarcoma | | Vimentin+, S100+ | | NA | | Complete resection | | NA | |
| 107 | Chiyo, M. et al^106^ | 13/F | Chest pain | 18.3 | Anterior mediastinum | | ML | | NA | | NA | | Complete resection | | NED/35 mo | |
| 108 | Munden, R. F. et al^107^ | 56/M | NA | NA | Mediastinum | | WDL | | NA | | NA | | NA | | NA | |
| 109 | Iqbal, M. et al^108^ | 78/M | Chest pain | 11.8 | Thorax | | Liposarcoma | | NA | | NA | | Complete resection | | NA | |
| 110 | Aubert, A. et al^109^ | 25/F | Chest pain | 4.8 | Mediastinum | | ML | | NA | | NA | | Incomplete resection +CT | | DOD/24 mo | |
| 111 | Aoki, T. et al^110^ | 49/M | NA | 19 | Posterior mediastinum | | WDL | | NA | | NA | | Complete resection | | NED/6 mo | |
| 112 | Eisenstat, R. et al^111^ | 56/F | Dyspnea | NA | Anterior mediastinum | | PL | | NA | | NA | | Biopsy | | NA | |
| 113 | Jung, J. I. et al^112^ | 37/F | Asymptomatic | 17 | Anterior mediastinum | | ML | | NA | | NA | | NA | | NA | |
| 114 | Gomez-Roman, J. J. et al^113^ | 76/M | Cough | 14.5 | Anterior mediastinum | | Lipo-LMS | | Vimentin+, SMA+, Desmin+, S100+ | | NA | | Complete resection | | Died of sepsis | |
| 115 | Krygier, G. et al^114^ | 49/M | Chest pain, dyspnea | 11.5 | Lung | | PL | | Vimentin+, S100+ | | NA | | Complete resection +RT | | DOD/ 8mo | |
| 116 | Ali, S. Z. et al^115^ | 3/M | NA | NA | Thorax | | PL | | NA | | NA | | RT+CT | | DOD/24 mo | |
| 117 | Chung, C. et al^116^ | 70/M | NA | NA | Anterior mediastinum | | Liposarcoma | | NA | | NA | | NA | | NA | |
| 118 | Attal, H. et al^117^ | 66/M | NA | NA | Anterior mediastinum | | ML | | NA | | NA | | NA | | NA | |
| 119 | Batouk, A. A. et al^118^ | 55/F | Chest pain, dyspnea | 10 | Right thorax | | WDL | | NA | | NA | | NA | | NA | |
| 120 | Mikkilineni, R. S. et al^119^ | 17/M | Chest pain, dyspnea | 30 | Posterior mediastinum | | ML | | NA | | NA | | Complete resection +CT | | DOD/ 9 mo | |
| 121 | Wong, William W. et al^120^ | 38/M | Chest pain, dyspnea | NA | Right thorax | | ML | | NA | | NA | | Complete resection +CT | | NED/5 mo | |
| 122 | Grewal, R. G. et al^121^ | 75/F | Dyspnea | 3 | Anterior mediastinum | | WDL | | NA | | NA | | Incomplete resection +RT | | AWD/ 60mo | |
| 123 | Carroll, F. et al^122^ | 23/F | Dyspnea | 29 | Left thorax | | Liposarcoma | | NA | | NA | | NA | | NA | |
| 124 | Sheppard, M. N. et al^123^ | 18/F | Cough, dyspnea | NA | Lung | | Liposarcoma | | NA | | NA | | Incomplete resection | | Died in operation | |
| 125 | Sachs, B. A. et al^124^ | 40/M | NA | 9 | Anterior mediastinum | | ML | | Vimentin+, S100+ | | NA | | Complete resection | | NA | |
| 126 | Downes, K. A.^125^ | 55/F | NA | 31 | Mediastinum | | PL | | NA | | NA | | NA | | DOD/2 mo | |
| 127 | Evans, H. L. et al^126^ | 45/F | Cough | 12 | Mediastinum | | Lipo-LMS | | NA | | NA | | Complete resection +RT+CT | | AWD/60 mo | |
| 128 | Evans, H. L. et al^127^ | 45/M | Arm weakness | 10 | Mediastinum | | DDL | | NA | | NA | | Complete resection | | NED/12 mo | |
| 129 | Huang, H. Y. et al^128^ | 39/M | NA | NA | Anterior mediastinum | | PL | | NA | | NA | | NA | | NA | |
| 130 | Matsubara, H. et al^129^ | 58/M | Dyspnea | NA | Mediastinum | | WDL | | NA | | NA | | Complete resection | | Recurrence at 240 mo, resection/ NED, 276 mo | |
| 131 | Miura, K. et al^130^ | 45/F | Asymptomatic | 12.7 | Middle mediastinum | | DDL | | NA | | NA | | Complete resection | | Recurrence at 51 mo, resection/ NED 60 mo | |
| 132 | Miura, K. et al^130^ | 62/F | Asymptomatic | 12 | Superior mediastinum | | DDL | | NA | | NA | | Complete resection | | Recurrence at 21 mo, RT/ AED, 28 mo | |
| 133 | Miura, K. et al^130^ | 84/M | Asymptomatic | 6.6 | Anterior mediastinum | | DDL | | MDM2 +, CDK4+ | | NA | | Complete resection | | Recurrence at 27, resection/ NED 40mo | |
| 134 | Miura, K. et al^130^ | 75/M | Asymptomatic | 20 | Posterior mediastinum | | DDL | | NA | | NA | | Complete resection | | NED/ 3 mo | |
| 135 | Miura, K. et al^130^ | 78/M | dyspnea | 11 | Middle mediastinum | | DDL | | NA | | NA | | CT | | DOD/ 0.5 mo | |
| 136 | Weissferdt, A. et al ^131^ | 52/F | Shortness of breath | 35 | Anterior mediastinum | | Lipo-LMS | | SMA+, Desmin+ | | NA | | Complete resection | | DOD/ 60 mo | |
| 137 | Weissferdt, A. et al ^131^ | 68/F | Shortness of breath | 13 | Anterior mediastinum | | WDL | | Desmin+,Myoglobin+ | | NA | | Complete resection | | NED/ 16 mo | |
| 138 | Okby, N. T. et al^132^ | 45/F | bronchitis | 16 | Pleural Cavity | | ML | | Vimentin+ | | NA | | Complete resection+CT | | DOD/7 mo | |
| 139 | Okby, N. T. et al^132^ | 73/M | Asymptomatic | NA | Pleural Cavity | | ML | | NA | | NA | | Incomplete resection | | DOD/9 mo | |
| 140 | Okby, N. T. et al^132^ | 67/M | Asymptomatic | 18.5 | Pleural Cavity | | WDL(sclerotic) | | NA | | NA | | NA | | Die of unrelated reason/16 mo | |
| 141 | Okby, N. T. et al^132^ | 80/M | Asymptomatic | 20 | Pleural Cavity | | ML | | NA | | NA | | Complete resection | | NA | |
| 142 | Blanco, María Jesús et al^133^ | 63/M | Asymptomatic | 30 | Mediastinum | | Lipo-LMS | | Actin+, desmin+ | | NA | | Complete resection | | NA | |
| 143 | Rossi, G. et al^134^ | 60/F | Dyspnea | 5 | Lung | | WDL(lipoma-like) | | S100+ | | NA | | Complete resection | | NED/ 84 mo | |
| 144 | Uchikov, A. et al^135^ | 49/M | Cough, chest pain | 2 | Lung | | ML | | Vimentin+, S100+ | | NA | | Complete resection | | NED/12 mo | |
| 145 | Son, C. et al^136^ | 24/F | Cough | 3.5 | Lung | | ML | | Vimentin+, S100+ | | *DDIT3-*(FISH) | | Complete resection | | NED/20 mo | |
| 146 | Alaggio, R. et al^137^ | 18/M | NA | NA | Mediastinum | | MPL | | NA | | NA | | NA | | DOD/8 mo | |
| 147 | Alaggio, R. et al^137^ | 18/F | NA | NA | Mediastinum | | MPL | | NA | | NA | | NA | | DOD/ 12 mo | |
| 148 | Alaggio, R. et al^137^ | 22/M | NA | NA | Mediastinum | | MPL | | NA | | NA | | NA | | NA | |
| 149 | Alaggio, R. et al^137^ | NA/F | NA | NA | Mediastinum | | MPL | | NA | | NA | | NA | | NA | |
| 150 | Alaggio, R. et al^137^ | 14/M | NA | NA | Mediastinum | | MPL | | NA | | NA | | NA | | DOD/ 36 mo | |
| 151 | Folpe, A. L. et al^138^ | NA/M | NA | NA | Mediastinum | | Lipo-LMS | | NA | | NA | | NA | | NA | |
| 152 | Folpe, A. L. et al^138^ | 54/M | NA | NA | Lung | | Lipo-LMS | | NA | | NA | | NA | | AWD/ 84 mo | |

Abbreviation: M, male; F, female; NA, not available; WDL, well-differentiated liposarcoma; DDL, dedifferentiated liposarcoma; ML, myxoid liposarcoma; PL, pleomorphic liposarcoma; M-PL, myxoid pleomorphic liposarcoma; Lipo-LMS: lipoleiomyosarcoma; IHC, immunohistochemistry; “+” positive, “-” negative; FISH, fluorescence in situ hybridization; RT, radiotherapy; CT, chemotherapy, NED, no evidence of disease; AWD, alive with disease; DFU, died from unrelated reasons; DOD, died of disease; mo, month.

1. Suzuki T, Sato T,Hasumi T. Resection of a giant mediastinal atypical lipomatous tumor involving the esophagus. J Surg Case Rep. (2021) 2021: rjaa561. DOI: 10.1093/jscr/rjaa561.

2. Alhames S,Ghabally M. Enbloc resection of the largest thymic liposarcoma: A case report with literature review. Ann Med Surg (Lond). (2020) 59: 204-206. DOI: 10.1016/j.amsu.2020.09.048.

3. Gaikwad NM, Srikrishna SV,Srikanth K. A rare case of a giant anterior mediastinal liposarcoma. Indian J Thorac Cardiovasc Surg. (2020) 36: 148-150. DOI: 10.1007/s12055-019-00871-6.

4. Wang L, Lv J, Lin R, Li X, Cheng X,Xin S. Mediastinal liposarcoma masquerading as penetrating aortic ulcer in the descending aorta: a case report. Cardiovasc Diagn Ther. (2020) 10: 888-891. DOI: 10.21037/cdt-20-287.

5. Furlan K, Miller I, Rohra P, Mir F, Ocampo Gonzalez FA,Gattuso P. Well-differentiated liposarcoma primary from thymic stroma: Case report and literature review. Exp Mol Pathol. (2020) 116: 104517. DOI: 10.1016/j.yexmp.2020.104517.

6. Iwamoto N, Matsuura Y, Ninomiya H, Ichinose J, Nakao M, Ishikawa Y*, et al.* An extremely rare case of rapidly growing mediastinal well-differentiated liposarcoma with a sclerosing variant: a case report. Surg Case Rep. (2020) 6: 158. DOI: 10.1186/s40792-020-00928-4.

7. Kang LH, Hwang CS,Yoon SH. Primary pleural liposarcoma combined spindle cell lipoma of the lung. Thorac Cancer. (2020) 11: 2059-2062. DOI: 10.1111/1759-7714.13495.

8. Ye YW, Liao MY, Mou ZM, Shi XX,Xie YC. Thoracoscopic resection of a huge esophageal dedifferentiated liposarcoma: A case report. World J Clin Cases. (2020) 8: 1698-1704. DOI: 10.12998/wjcc.v8.i9.1698.

9. Chen HG, Zhang K, Wu WB, Wu YH, Zhang J, Gu LJ*, et al.* Combining surgery with (125)I brachytherapy for recurrent mediastinal dedifferentiated liposarcoma: A case report and review of literature. World J Clin Cases. (2020) 8: 939-945. DOI: 10.12998/wjcc.v8.i5.939.

10. Mei X, Li M,Xia Y. A huge mediastinal, well-differentiated liposarcoma with heterogenous smooth muscle differentiation: a case report. Int J Clin Exp Pathol. (2019) 12: 2763-2766. DOI:

11. Boatright C, Walker CM, Donald J, Cui W,Nagji AS. Incidental dedifferentiated mediastinal liposarcoma on F-18-fluciclovine PET/CT. Clin Imaging. (2020) 59: 21-24. DOI: 10.1016/j.clinimag.2019.08.004.

12. Prabhakar N, Vaiphei K, Vishwajeet V, Ramamoorthy E, Gorsi U, Dhooria S*, et al.* Primary pleural liposarcoma: A rare entity. Lung India. (2019) 36: 438-440. DOI: 10.4103/lungindia.lungindia_246_18.

13. Zhang M, Zhang S, Shi H, Li W,Wei Z. Resection of a huge mediastinal well-differentiated liposarcoma involving left thoracic cavity. J Cardiothorac Surg. (2019) 14: 148. DOI: 10.1186/s13019-019-0965-0.

14. Ishtiaq R, Naeem A,Ratnani I. Thoracic Liposarcoma In An End Stage Renal Disease Patient. J Ayub Med Coll Abbottabad. (2019) 31: 286-289. DOI:

15. Ashraf U, Dudekula RA, Roy S, Burack J, Malik S,Khaja M. Recurrent intrathoracic dedifferentiated liposarcoma: A case report and literature review. Respir Med Case Rep. (2019) 26: 281-284. DOI: 10.1016/j.rmcr.2019.02.016.

16. Matsukuma S, Oshika Y, Utsumi Y, Obara K, Tanimoto T, Katsurada Y*, et al.* Pleural dedifferentiated liposarcoma: A case report. Mol Clin Oncol. (2019) 10: 132-136. DOI: 10.3892/mco.2018.1757.

17. Kandakure PR, Kambhampati S, Katta Y, Timanwar A,Lakka VK. Giant bilateral posterior mediastinal liposarcoma excision. Indian J Thorac Cardiovasc Surg. (2019) 35: 91-93. DOI: 10.1007/s12055-018-0703-6.

18. Yang YS, Bai CY, Li ZC, Li WJ,Li Y. Giant primary liposarcoma of the anterior mediastinum: A case report. Medicine (Baltimore). (2018) 97: e12873. DOI: 10.1097/MD.0000000000012873.

19. Soeroso NN, Pradana A, Djaka M, Ayudika M, Ngadimin S,Soeroso L. An unusual case of recurrent huge primary mediastinal dedifferentiated liposarcoma. Int J Surg Case Rep. (2018) 50: 140-143. DOI: 10.1016/j.ijscr.2018.07.037.

20. Chen C, Chen M, Liu W, Yuan Y,Yu F. Successful removal of giant mediastinal lipoma and liposarcoma involving both chest cavities: Two case reports. Medicine (Baltimore). (2018) 97: e11806. DOI: 10.1097/MD.0000000000011806.

21. Kim SY, Froelich JJ, Dawson H, Peters AA, Tappero C,Heverhagen JT. Inflammatory calcified de-differentiated liposarcoma of the anterior mediastinum. ANZ J Surg. (2019) 89: 1326-1327. DOI: 10.1111/ans.14697.

22. Pui WC, Ling WHY, Najah M,Soon SY. Successful resection of a giant thoracic myxoid liposarcoma. Asian Cardiovasc Thorac Ann. (2018) 26: 410-412. DOI: 10.1177/0218492318772763.

23. Nguyen DC, Olatubosun O, Yu W, Loor G,Burt BM. Giant Mediastinal Liposarcoma: A Rare Yet Distinct Clinical Entity. Ann Thorac Surg. (2018) 106: e117-e119. DOI: 10.1016/j.athoracsur.2018.03.018.

24. Pusiol T, Piscioli I, Rondoni V,Scialpi M. Intrathoracic liposarcoma: Case report with emphasis to histogenesis and site of origin classification problems. Lung India. (2018) 35: 186-187. DOI: 10.4103/lungindia.lungindia_332_17.

25. Takenaka M, Ichiki Y, Taira A,Tanaka F. Extended surgery using anterior mediastinal tracheostomy for recurrent mediastinal liposarcoma†. European Journal of Cardio-Thoracic Surgery. (2018) 54: 397-399. DOI: 10.1093/ejcts/ezy021.

26. Tong JJ, Li XJ, Li GX,Qian GQ. Lipoleiomyosarcoma of the posterior mediastinum. QJM. (2018) 111: 191-192. DOI: 10.1093/qjmed/hcx256.

27. Galli A, Giordano L, Muriana P, Bandiera A, Negri G, Zannini P*, et al.* Multidisciplinary management of a giant cervico-mediastinal liposarcoma: A case report and literature review. Ear Nose Throat J. (2017) 96: E10-E13. DOI: 10.1177/014556131709601213.

28. Weaver HL, Preston SD, Wong HH, Jani P,Coonar AS. Surgical resection of a massive primary mediastinal liposarcoma with cervical extension. Ann R Coll Surg Engl. (2018) 100: e22-e27. DOI: 10.1308/rcsann.2017.0163.

29. Sugiura Y, Hashizume T, Fujimoto H,Nemoto E. A giant mediastinal liposarcoma weighing 3500g resected with clam shell approach, a case report with review of literature. Int J Surg Case Rep. (2017) 41: 292-295. DOI: 10.1016/j.ijscr.2017.10.055.

30. Edagawa M, Haratake N, Shimamatsu S, Toyozawa R, Nosaki K, Hirai F*, et al.* Surgical resection of a well-differentiated inflammatory liposarcoma of the middle mediastinum: a case report. J Thorac Dis. (2017) 9: E689-E693. DOI: 10.21037/jtd.2017.07.90.

31. Khan MH, Kashif R, Rahim Khan HA,Fatimi SH. Myxoid Liposarcoma Originating In The Anterior Mediastinum. J Ayub Med Coll Abbottabad. (2016) 28: 818-820. DOI:

32. Wang F, Kiryu S, Li L, Wang Q, Li D,Zhang L. Resectable primary pleural myxoid liposarcoma with a pedicle: report of a rare case and literature review. J Thorac Dis. (2017) 9: E183-E187. DOI: 10.21037/jtd.2017.03.29.

33. Toda M, Izumi N, Tsukioka T, Komatsu H, Okada S, Hara K*, et al.* Huge mediastinal liposarcoma resected by clamshell thoracotomy: a case report. Surg Case Rep. (2017) 3: 16. DOI: 10.1186/s40792-017-0291-5.

34. Huang W,Jiang GN. Resection of giant mediastinal liposarcoma via ' dash, vertical shape' incision. J Surg Case Rep. (2017) 2017: DOI: 10.1093/jscr/rjw219.

35. Zhao C, Zhang F, Zhang X, Tu S, Wu Z, Li X*, et al.* Recurrent primary mediastinal liposarcoma: A case report. Oncol Lett. (2016) 11: 3782-3784. DOI: 10.3892/ol.2016.4453.

36. Lin F, Pu Q, Ma L, Liu C, Mei J, Liao H*, et al.* Successful resection of a huge mediastinal liposarcoma extended to the bilateral thorax. Thorac Cancer. (2016) 7: 373-376. DOI: 10.1111/1759-7714.12285.

37. Arava S, Breta M, Madan K, Nath D, Mehta S,Jain D. Sclerosing liposarcoma of the anterior mediastinum: An unusual case. Indian J Pathol Microbiol. (2016) 59: 69-71. DOI: 10.4103/0377-4929.178226.

38. Li YZ, Chae KJ, Jin GY,Park HS. A Well-Differentiated Mediastinal Liposarcoma from the Posterior Mediastinum with Separated Solid and Lipomatous Regions: A Case Report. Am J Med. (2016) 129: e71-72. DOI: 10.1016/j.amjmed.2016.01.028.

39. Biswas A, Urbine D, Prasad A, Papierniak ES, Weber M, Malhotra P*, et al.* Patient With Slow-Growing Mediastinal Mass Presents With Chest Pain and Dyspnea. Chest. (2016) 149: e17-23. DOI: 10.1016/j.chest.2015.10.043.

40. Hamanaka K, Ohashi M,Nakamura T. Primary mediastinal dedifferentiated liposarcoma resected by lateral thoracotomy with video-assisted thoracoscopic surgery. J Surg Case Rep. (2016) 2016: DOI: 10.1093/jscr/rjv163.

41. Mani VR, Ofikwu G,Safavi A. Surgical resection of a giant primary liposarcoma of the anterior mediastinum. J Surg Case Rep. (2015) 2015: DOI: 10.1093/jscr/rjv126.

42. Harth S, Litzlbauer HD, Behrens CB, Roller FC, Gamerdinger U, Burchert D*, et al.* Dedifferentiated Liposarcoma of the Anterior Mediastinum: A Rare Case. Rofo. (2016) 188: 95-97. DOI: 10.1055/s-0035-1553367.

43. Ma J, Zhang HM, Zhang LW, Zheng MW,Yu M. Primary mediastinal giant liposarcoma with smooth muscle and neural differentiation: A case report. Oncol Lett. (2015) 9: 2667-2669. DOI: 10.3892/ol.2015.3103.

44. Kawamura T, Tomii K, Takahashi Y, Okada A, Demizu Y, Fuwa N*, et al.* Recurrence of a mediastinal liposarcoma 20 years after surgery: A case of carbon ion radiotherapy resulting in fatal tracheoesophageal fistula. Respir Investig. (2015) 53: 170-172. DOI: 10.1016/j.resinv.2015.02.002.

45. Arraras-Martinez MJ, Rieger-Reyes C, Panadero-Paz C, Landa-Oviedo HS,Garcia-Tirado J. Giant primary mediastinal liposarcoma: A rare cause of atrial flutter. Asian Cardiovasc Thorac Ann. (2015) 23: 1121-1124. DOI: 10.1177/0218492315589201.

46. Rena O, Davoli F, Pia F, Roncon A, Papalia E,Casadio C. Giant Cervico-mediastinal Well-differentiated Liposarcoma. Heart Lung Circ. (2015) 24: e112-114. DOI: 10.1016/j.hlc.2015.02.015.

47. Fujimoto R, Sato M, Miyata R, Minakata K, Omasa M, Kubo T*, et al.* Successful resection of recurrent mediastinal liposarcoma using preoperative evaluation of organ invasion by four-dimensional computed tomography. Gen Thorac Cardiovasc Surg. (2016) 64: 488-491. DOI: 10.1007/s11748-014-0510-y.

48. Asaka S, Yoshida K, Hashizume M,Ito K. A mediastinal liposarcoma resected using a double approach with a thoracoscope. Thorac Cardiovasc Surg Rep. (2013) 2: 46-49. DOI: 10.1055/s-0033-1358605.

49. Carrillo BJ, Navarrete C, Lopez Arias MA,Pelaez M. Primary pleural liposarcoma, pleomorphic variant. J Thorac Dis. (2014) 6: E166-168. DOI: 10.3978/j.issn.2072-1439.2014.07.30.

50. Chen G, Qiu X, Liu Y, Qiao Y, Shi T, Chen J*, et al.* Primary mediastinal pleomorphic liposarcoma involving the superior vena cava. Thorac Cancer. (2014) 5: 265-270. DOI: 10.1111/1759-7714.12084.

51. Longano A, DuGuesclin A,Mitchell C. Primary dedifferentiated liposarcoma of the lung with rhabdomyoblastic and chrondroblastic differentiation. Histopathology. (2015) 67: 923-925. DOI: 10.1111/his.12410.

52. Fukuhara S, Dimitrova KR, Geller CM, Hoffman DM, Ko W,Tranbaugh RF. Progressive dyspnea in patient with large mediastinal mass. J Cardiothorac Surg. (2014) 9: 6. DOI: 10.1186/1749-8090-9-6.

53. Hirano Y, Yamamoto H, Ichimura K, Toyooka S,Miyoshi S. Surgical resection of a massive primary mediastinal liposarcoma using clamshell incision combined with lower median sternotomy: report of a case. Ann Thorac Cardiovasc Surg. (2014) 20 Suppl: 606-608. DOI: 10.5761/atcs.cr.13.02263.

54. Billè A, Garofalo G, Leo F,Pastorino U. Giant liposarcoma elongating mediastinal vessels with intrathoracic inferior vena cava replacement. European Journal of Cardio-Thoracic Surgery. (2013) 44: 570-572. DOI: 10.1093/ejcts/ezt149.

55. Zhou LF, Zhang SJ, Shao YF, Liu Y, Hu Z, Li MM*, et al.* A unique life-threatening mediastinal liposarcoma mimicking pleural effusion. Am J Respir Crit Care Med. (2012) 186: 106. DOI: 10.1164/rccm.201109-1676IM.

56. Kashu Y, Yukumi S, Tsunooka N, Tanigawa K, Arakane M, Nakagawa H*, et al.* Successful resection of a massive mediastinal liposarcoma that rapidly extended into the entire left thoracic cavity: report of a case. Surg Today. (2012) 42: 68-71. DOI: 10.1007/s00595-011-0013-8.

57. Matsuo T, Takamori S, Hayabuchi N, Fumihiko M, Kashihara M, Yoshiyama K*, et al.* Giant liposarcoma occupying most of the hemi-thorax and resected in the supine position: report of a rare case. Kurume Med J. (2011) 58: 63-65. DOI: 10.2739/kurumemedj.58.63.

58. Decker JR, de Hoyos AL,Decamp MM. Successful thoracoscopic resection of a large mediastinal liposarcoma. Ann Thorac Surg. (2011) 92: 1499-1501. DOI: 10.1016/j.athoracsur.2011.02.051.

59. Elsayed H,Gosney J. A massive pleural-based tumour: the challenge of diagnosis. Rev Port Pneumol. (2011) 17: 275-277. DOI: 10.1016/j.rppneu.2011.06.001.

60. Shoji F, Taketomi A, Yano T,Maehara Y. Intraoperative radiofrequency ablation in an open thoracotomy setting for the new treatment of mediastinal liposarcoma: report of a case. Surg Today. (2011) 41: 992-994. DOI: 10.1007/s00595-010-4380-3.

61. Sivaraman A, Jaikaran GK, Suresh RV,Sashank RK. Giant thymoliposarcoma presented like thymolipoma. Eur J Cardiothorac Surg. (2011) 39: e146-147. DOI: 10.1016/j.ejcts.2011.01.028.

62. Wiedemann D, Schistek R, Gassner E,Antretter H. Mediastinal liposarcoma. J Card Surg. (2011) 26: 162-164. DOI: 10.1111/j.1540-8191.2010.01174.x.

63. Taki K, Watanabe M, Iwagami S, Nagai Y, Iwatsuki M, Ishimoto T*, et al.* Giant liposarcoma of the posterior mediastinum and retroperitoneum. BMJ Case Rep. (2011) 2011: DOI: 10.1136/bcr.06.2011.4341.

64. Okuno M, Kawashima M, Miura K, Kadota E, Goto S,Kato M. Resection of giant mediastinal liposarcoma using the hemiclamshell incision. Gen Thorac Cardiovasc Surg. (2010) 58: 654-656. DOI: 10.1007/s11748-010-0584-0.

65. Lin YY,Su WF. Images in clinical medicine. Liposarcoma of the anterior mediastinum and neck. N Engl J Med. (2010) 363: 864. DOI: 10.1056/NEJMicm0912793.

66. Saeed M, Plett S, Kim GE, Daldrup-Link H,Courtier J. Radiological-pathological correlation of pleomorphic liposarcoma of the anterior mediastinum in a 17-year-old girl. Pediatr Radiol. (2010) 40 Suppl 1: S68-70. DOI: 10.1007/s00247-010-1797-1.

67. Liu LG, Wei X,Pan TC. A giant primary myxoid liposarcoma of the posterior mediastinum. Chin Med J (Engl). (2010) 123: 1818-1820. DOI:

68. Dagli AF, Pehlivan S,Ozercan MR. Pleural liposarcoma mimicking carcinoma in pleural effusion cytology: a case report. Acta Cytol. (2010) 54: 601-604. DOI: 10.1159/000325185.

69. Anand Rajan KD, Subbarao KC, Agarwala S,Gupta SD. Mediastinal liposarcoma of mixed type in childhood: a report of a case with unusual histologic features. Indian J Pathol Microbiol. (2010) 53: 525-528. DOI: 10.4103/0377-4929.68297.

70. Konno S, Oizumi S, Shinagawa N, Kikuchi E, Konishi J, Ito K*, et al.* Primary mediastinal liposarcoma, with 6 years of follow-up to autopsy, revealed histopathological features of primary and metastatic lesions. Intern Med. (2010) 49: 771-775. DOI: 10.2169/internalmedicine.49.2974.

71. Gethin-Jones TL, Evans NR, 3rd,Morse CR. Surgical management of mediastinal liposarcoma extending from hypopharynx to carina: case report. World J Surg Oncol. (2010) 8: 13. DOI: 10.1186/1477-7819-8-13.

72. Chaput-Dugas ME, Chughtai T, Liberman M, Duranceau A, Martin J, Barkat F*, et al.* Successful resection of a large incidentally found primary mediastinal liposarcoma. J Surg Case Rep. (2010) 2010: 1. DOI: 10.1093/jscr/2010.8.1.

73. Thomaz FB, Marchiori E, Guimaraes AN, de Magalhaes IF, Magalhaes FV, Goncalves LP*, et al.* Primary mediastinal liposarcoma - computed tomography and pathological findings: a case report. Cases J. (2009) 2: 8703. DOI: 10.4076/1757-1626-2-8703.

74. Gasiorowski L, Dyszkiewicz W,Piwkowski CT. An unusual case of giant primary mediastinal liposarcoma. Thorac Cardiovasc Surg. (2009) 57: 247-248. DOI: 10.1055/s-2008-1039056.

75. Coulibaly B, Bouvier C, Payan MJ,Thomas P. Recurrent dedifferentiated liposarcoma of mediastinum involving lung and pleura. Interact Cardiovasc Thorac Surg. (2009) 9: 741-742. DOI: 10.1510/icvts.2009.209494.

76. Berry MF, Sporn TA, Moore JO,D'Amico TA. Giant thoracic liposarcoma treated with induction chemotherapy followed by surgical resection. J Thorac Oncol. (2009) 4: 768-769. DOI: 10.1097/JTO.0b013e31819e77ff.

77. Fukai R, Fukumura Y,Suzuki K. A dedifferentiated liposarcoma of the anterior mediastinum. Int J Clin Oncol. (2009) 14: 174-177. DOI: 10.1007/s10147-008-0819-6.

78. Shoji T, Sonobe M, Okubo K, Wada H, Bando T,Date H. Giant primary liposarcoma of the chest. General Thoracic and Cardiovascular Surgery. (2009) 57: 159-161. DOI: 10.1007/s11748-008-0329-5.

79. Achir A, Ouadnouni Y, Smahi M, Bouchikh M, Msougar Y,Benosman A. Primary pulmonary liposarcoma--a case report. Thorac Cardiovasc Surg. (2009) 57: 119-120. DOI: 10.1055/s-2006-955885.

80. Alloubi I, Boubia S,Ridai M. Liposarcoma of the pleural cavity. Thorac Cardiovasc Surg. (2008) 56: 438-439. DOI: 10.1055/s-2007-965711.

81. Greif J, Marmor S, Merimsky O, Kovner F,Inbar M. Primary liposarcoma of the mediastinum. Sarcoma. (1998) 2: 205-207. DOI: 10.1080/13577149877993.

82. Barbetakis N, Samanidis G, Samanidou E, Kirodimos E, Kiziridou A, Bischiniotis T*, et al.* Primary mediastinal liposarcoma: a case report. J Med Case Rep. (2007) 1: 161. DOI: 10.1186/1752-1947-1-161.

83. Benchetritt M, Hofman V, Venissac N, Brennetot C, Italiano A, Aurias A*, et al.* Dedifferentiated liposarcoma of the pleura mimicking a malignant solitary fibrous tumor and associated with dedifferentiated liposarcoma of the mediastinum: usefulness of cytogenetic and molecular genetic analyses. Cancer Genet Cytogenet. (2007) 179: 150-155. DOI: 10.1016/j.cancergencyto.2007.09.006.

84. Goldsmith P,Papagiannopoulos K. Pleural myxoid liposarcoma: features of 2 cases and associated literature review. J Cardiothorac Surg. (2007) 2: 48. DOI: 10.1186/1749-8090-2-48.

85. Raghavan R, Raghuram P, Parekh PV,Kurien JM. Posterior mediastinal liposarcoma simulating a lung mass: an unusual case report. Cancer Imaging. (2007) 7: 141-144. DOI: 10.1102/1470-7330.2007.0021.

86. Munjal K, Pancholi V, Rege J, Munjal S, Bhandari V,Nahar R. Fine needle aspiration cytology in mediastinal myxoid liposarcoma: a case report. Acta Cytol. (2007) 51: 456-458. DOI: 10.1159/000325766.

87. Romero-Guadarrama MB, Jiménez-Becerra S, Durán-Padilla MA, Santiago-Prieto AC, Cruz-Ortiz H,Novelo-Retana V. Mediastinal pleomorphic liposarcoma diagnosed by fine needle aspiration biopsy: a case report. Acta Cytol. (2007) 51: 440-442. DOI: 10.1159/000325763.

88. Marulli G, Rea F, Feltracco P, Calabrese F, Giacometti C, Rizzardi G*, et al.* Successful resection of a giant primary liposarcoma of the posterior mediastinum. J Thorac Oncol. (2007) 2: 453-455. DOI: 10.1097/01.JTO.0000268681.10367.cf.

89. Peng C, Zhao X, Dong X,Jiang X. Liposarcoma of the pleural cavity: a case report. J Thorac Cardiovasc Surg. (2007) 133: 1108-1109. DOI: 10.1016/j.jtcvs.2006.12.014.

90. Trahan S, Erickson-Johnson MR, Rodriguez F, Aubry MC, Cheville JC, Myers JL*, et al.* Formation of the 12q14-q15 amplicon precedes the development of a well-differentiated liposarcoma arising from a nonchondroid pulmonary hamartoma. Am J Surg Pathol. (2006) 30: 1326-1329. DOI: 10.1097/01.pas.0000213257.69478.2f.

91. Marolla A, Pardolesi A, Camplese P, Politi R,Sacco R. Giant posterior mediastinal liposarcoma invading the esophagus: a case report. Rays. (2006) 31: 17-19. DOI:

92. Hirai S, Hamanaka Y, Mitsui N, Uegami S,Matsuura Y. Surgical resection of primary liposarcoma of the anterior mediastinum. Ann Thorac Cardiovasc Surg. (2008) 14: 38-41. DOI:

93. Punpale A, Pramesh CS, Jambhekar N,Mistry RC. Giant mediastinal liposarcoma: a case report. Ann Thorac Cardiovasc Surg. (2006) 12: 425-427. DOI:

94. Minniti A, Montaundon M, Jougon J, Hourneau M, Begueret H, Laurent F*, et al.* Liposarcoma of the pleural cavity. An exceptional tumour. Monaldi Arch Chest Dis. (2005) 63: 170-172. DOI: 10.4081/monaldi.2005.637.

95. Ibe T, Otani Y, Shimizu K, Nakano T, Sano T,Morishita Y. Pulmonary pleomorphic liposarcoma. Jpn J Thorac Cardiovasc Surg. (2005) 53: 443-447. DOI: 10.1007/s11748-005-0082-y.

96. Loddenkemper C, Pérez-Canto A, Leschber G,Stein H. Primary dedifferentiated liposarcoma of the lung. Histopathology. (2005) 46: 710-712. DOI: 10.1111/j.1365-2559.2005.02041.x.

97. Takanami I,Imamura T. Dedifferentiated liposarcoma of the pleura: report of a case. Surg Today. (2005) 35: 313-316. DOI: 10.1007/s00595-004-2940-0.

98. Ohta Y, Murata T, Tamura M, Sato H, Kurumaya H,Katayanagi K. Surgical resection of recurrent bilateral mediastinal liposarcoma through the clamshell approach. Ann Thorac Surg. (2004) 77: 1837-1839. DOI: 10.1016/s0003-4975(03)01242-6.

99. Noji T, Morikawa T, Kaji M, Ohtake S,Katoh H. Successful resection of a recurrent mediastinal liposarcoma invading the pericardium: report of a case. Surg Today. (2004) 34: 450-452. DOI: 10.1007/s00595-003-2719-8.

100. Said M, Migaw H, Hafsa C, Braham R, Golli M, Moussa A*, et al.* Imaging features of primary pulmonary liposarcoma. Australas Radiol. (2003) 47: 313-317. DOI: 10.1046/j.1440-1673.2003.01198.x.

101. Kim SH, Choi YJ, Kim HJ,Yang WI. Liposarcoma with meningothelial-like whorls. Report of four cases showing diverse histologic findings and behavior. Yonsei Med J. (2003) 44: 392-400. DOI: 10.3349/ymj.2003.44.3.392.

102. Paci M, De Franco S, Cavazza A, Annessi V, Ferrari G, Falco F*, et al.* Well-differentiated giant "lipoma-like" liposarcoma of the posterior mediastinum: a case report. Chir Ital. (2003) 55: 101-104. DOI:

103. Mase T, Kawawaki N, Narumiya C, Aoyama T, Kato S,Nagata Y. Primary liposarcoma of the mediastinum. Jpn J Thorac Cardiovasc Surg. (2002) 50: 252-255. DOI: 10.1007/bf03032155.

104. Sakamaki Y, Miyoshi S, Minami M, Tanaka H, Inada K,Matsuda H. Mediastinal liposarcoma appearing as a tumor arising in the esophageal wall. Jpn J Thorac Cardiovasc Surg. (2001) 49: 679-681. DOI: 10.1007/bf02912480.

105. Kara M, Ozkan M, Dizbay Sak S,Kavukçu ST. Successful removal of a giant recurrent mediastinal liposarcoma involving both hemithoraces. Eur J Cardiothorac Surg. (2001) 20: 647-649. DOI: 10.1016/s1010-7940(01)00848-x.

106. Chiyo M, Fujisawa T, Yasukawa T, Shiba M, Shibuya K, Sekine Y*, et al.* Successful resection of a primary liposarcoma in the anterior mediastinum in a child: report of a case. Surg Today. (2001) 31: 230-232. DOI: 10.1007/s005950170174.

107. Munden RF, Nesbitt JC, Kemp BL, Chasen MH,Whitman GJ. Primary liposarcoma of the mediastinum. AJR Am J Roentgenol. (2000) 175: 1340. DOI: 10.2214/ajr.175.5.1751340.

108. Iqbal M, Posen J, Bhuiya TA, Lackner RP, Steinberg HN,Rossoff LJ. Lymphocyte-rich pleural liposarcoma mimicking pericardial cyst. J Thorac Cardiovasc Surg. (2000) 120: 610-612. DOI: 10.1067/mtc.2000.106971.

109. Aubert A, Chaffanjon P, Peoch M,Brichon PY. Chest wall implantation of a mediastinal liposarcoma after thoracoscopy. Ann Thorac Surg. (2000) 69: 1579-1580; discussion 1581. DOI: 10.1016/s0003-4975(00)01195-4.

110. Aoki T, Sugawara M, Yamato Y,Hayashi J. A surgical management of aortic insufficiency concomitant with mediastinal well-differentiated liposarcoma. Thorac Cardiovasc Surg. (1999) 47: 397-399. DOI: 10.1055/s-2007-1013184.

111. Eisenstat R, Bruce D, Williams LE,Katz DS. Primary liposarcoma of the mediastinum with coexistent mediastinal lipomatosis. AJR Am J Roentgenol. (2000) 174: 572-573. DOI: 10.2214/ajr.174.2.1740572.

112. Jung JI, Kim H, Kang SW,Park SH. Radiological findings in myxoid liposarcoma of the anterior mediastinum. Br J Radiol. (1998) 71: 975-976. DOI: 10.1259/bjr.71.849.10195015.

113. Gomez-Roman JJ,Val-Bernal JF. Lipoleiomyosarcoma of the mediastinum. Pathology. (1997) 29: 428-430. DOI: 10.1080/00313029700169475.

114. Krygier G, Amado A, Salisbury S, Fernandez I, Maedo N,Vazquez T. Primary lung liposarcoma. Lung Cancer. (1997) 17: 271-275. DOI: 10.1016/s0169-5002(97)00030-5.

115. Ali SZ, Teichberg S, Kahn E,Hajdu SI. Intrathoracic pleomorphic liposarcoma in a child: study of an unusual case. Pediatr Pathol Lab Med. (1996) 16: 99-105. DOI:

116. Chung C, Lu CC, Chang SC, Hsu WH,Perng RP. Mediastinal liposarcoma with local recurrence: a case report. Zhonghua Yi Xue Za Zhi (Taipei). (1996) 57: 70-73. DOI:

117. Attal H, Jensen J,Reyes CV. Myxoid liposarcoma of the anterior mediastinum. Diagnosis by fine needle aspiration biopsy. Acta Cytol. (1995) 39: 511-513. DOI:

118. Batouk AA, Albdah AM, Agina HA,Khan AR. Primary pleural liposarcoma. Ann Saudi Med. (1995) 15: 159-161. DOI: 10.5144/0256-4947.1995.159.

119. Mikkilineni RS, Bhat S, Cheng AW,Prevosti LG. Liposarcoma of the posterior mediastinum in a child. Chest. (1994) 106: 1288-1289. DOI: 10.1378/chest.106.4.1288.

120. Wong WW, Pluth JR, Grado GL, Schild SE,Sanderson DR. Liposarcoma of the Pleura. Mayo Clinic Proceedings. (1994) 69: 882-885. DOI: 10.1016/s0025-6196(12)61792-3.

121. Grewal RG, Prager K, Austin JH,Rotterdam H. Long term survival in non-encapsulated primary liposarcoma of the mediastinum. Thorax. (1993) 48: 1276-1277. DOI: 10.1136/thx.48.12.1276.

122. Carroll F, Kramer MD, Acinapura AJ, Tietjen PA, Wagner I, Oiseth S*, et al.* Pleural liposarcoma presenting with respiratory distress and suspected diaphragmatic hernia. Ann Thorac Surg. (1992) 54: 1212-1213. DOI: 10.1016/0003-4975(92)90102-a.

123. Sheppard MN. Primary liposarcoma of the lung in a young woman. Thorax. (1990) 45: 908. DOI: 10.1136/thx.45.11.908.

124. Sachs BA, Shahab N, Kaplan PA, Doll DC,Haider S. Conditions suggesting lymphoma: case 2. Mediastinal liposarcoma in a patient with previous testicular cancer. J Clin Oncol. (2005) 23: 3844-3846. DOI: 10.1200/JCO.2005.04.166.

125. Downes KA, Goldblum JR, Montgomery EA,Fisher C. Pleomorphic liposarcoma: a clinicopathologic analysis of 19 cases. Mod Pathol. (2001) 14: 179-184. DOI: 10.1038/modpathol.3880280.

126. Evans HL. Smooth muscle in atypical lipomatous tumors. A report of three cases. Am J Surg Pathol. (1990) 14: 714-718. DOI: 10.1097/00000478-199008000-00002.

127. Evans HL, Khurana KK, Kemp BL,Ayala AG. Heterologous elements in the dedifferentiated component of dedifferentiated liposarcoma. Am J Surg Pathol. (1994) 18: 1150-1157. DOI:

128. Huang HY,Antonescu CR. Epithelioid variant of pleomorphic liposarcoma: a comparative immunohistochemical and ultrastructural analysis of six cases with emphasis on overlapping features with epithelial malignancies. Ultrastruct Pathol. (2002) 26: 299-308. DOI: 10.1080/01913120290104575.

129. Matsubara H, Mizutani E, Okuwaki H, Nagasaka S, Miyauchi Y, Oyachi N*, et al.* Recurrent mediastinal liposarcoma twenty years after the initial operation: case report. Ann Thorac Cardiovasc Surg. (2007) 13: 407-409. DOI:

130. Miura K, Hamanaka K, Matsuoka S, Takeda T, Agatsuma H, Hyogotani A*, et al.* Primary mediastinal dedifferentiated liposarcoma: Five case reports and a review. Thorac Cancer. (2018) 9: 1733-1740. DOI: 10.1111/1759-7714.12888.

131. Weissferdt A,Moran CA. Lipomatous tumors of the anterior mediastinum with muscle differentiation: a clinicopathological and immunohistochemical study of three cases. Virchows Arch. (2014) 464: 489-493. DOI: 10.1007/s00428-014-1556-z.

132. Okby NT,Travis WD. Liposarcoma of the pleural cavity: clinical and pathologic features of 4 cases with a review of the literature. Arch Pathol Lab Med. (2000) 124: 699-703. DOI: 10.1043/0003-9985(2000)124<0699:lotpc>2.0.co;2.

133. Blanco MJ, Rezola F,Santana A. Lipoleiomyosarcoma of the Mediastinum in an Asymptomatic Male Patient. Archivos de Bronconeumología (English Edition). (2017) 53: 690. DOI: 10.1016/j.arbr.2017.10.009.

134. Rossi G, Cavazza A, Valli R, Torricelli P, Richeldi L, Rivasi F*, et al.* Atypical lipomatous tumour (lipoma-like well-differentiated liposarcoma) arising in a pulmonary hamartoma and clinically presenting with pneumothorax. Lung Cancer. (2003) 39: 103-106. DOI: 10.1016/s0169-5002(02)00393-8.

135. Uchikov A, Poriazova E, Zaprianov Z,Markova D. Low-grade pulmonary myxoid liposarcoma. Interact Cardiovasc Thorac Surg. (2005) 4: 402-403. DOI: 10.1510/icvts.2005.108332.

136. Son C, Choi PJ,Roh MS. Primary Pulmonary Myxoid Liposarcoma with Translocation t(12;16)(q13;p11) in a Young Female Patient: A Brief Case Report. Korean J Pathol. (2012) 46: 392-394. DOI: 10.4132/KoreanJPathol.2012.46.4.392.

137. Alaggio R, Coffin CM, Weiss SW, Bridge JA, Issakov J, Oliveira AM*, et al.* Liposarcomas in Young Patients A Study of 82 Cases Occurring in Patients Younger Than 22 Years of Age. American journal of surgical pathology. (2009) 33: 645-658. DOI: Doi 10.1097/Pas.0b013e3181963c9c.

138. Folpe AL,Weiss SW. Lipoleiomyosarcoma (well-differentiated liposarcoma with leiomyosarcomatous differentiation): a clinicopathologic study of nine cases including one with dedifferentiation. Am J Surg Pathol. (2002) 26: 742-749. DOI: 10.1097/01.pas.0000016311.09846.57.
